# β-Hairpin mimics containing a piperidine–pyrrolidine scaffold modulate the β-amyloid aggregation process preserving the monomer species†
†Electronic supplementary information (ESI) available: Computational methods and additional figures and tables. NMR additional data. Description of synthetic procedures and characterization of compounds. Experimental procedure for fluorescence-detected ThT binding assay; representative curves of ThT fluorescence assays. Experimental procedure for TEM studies, CE, and cellular evaluation. See DOI: 10.1039/c6sc03176e



**DOI:** 10.1039/c6sc03176e

**Published:** 2016-10-07

**Authors:** S. Pellegrino, N. Tonali, E. Erba, J. Kaffy, M. Taverna, A. Contini, M. Taylor, D. Allsop, M. L. Gelmi, S. Ongeri

**Affiliations:** a DISFARM-Sez. Chimica Generale e Organica “A. Marchesini” , Universitá degli Studi di Milano , via Venezian 21 , 20133 Milano , Italy . Email: sara.pellegrino@unimi.it; b Molécules Fluorées et Chimie Médicinale , BioCIS , Univ. Paris-Sud , CNRS , Université Paris Saclay , 5 rue Jean-Baptiste Clément , 92296 Châtenay-Malabry Cedex , France . Email: Sandrine.ongeri@u-psud.fr; c Protéines et Nanotechnologies en Sciences Séparatives , Institut Galien Paris-Sud , Univ. Paris-Sud , CNRS , Université Paris Saclay , 5 rue Jean-Baptiste Clément , 92296 Châtenay-Malabry Cedex , France; d Lancaster University , Division of Biomedical and Life Sciences , Faculty of Health and Medicine , Lancaster LA1 4YQ , UK

## Abstract

Alzheimer's disease is a neurodegenerative disorder linked to oligomerization and fibrillization of amyloid β peptides, with Aβ_1–42_ being the most aggregative and neurotoxic one. We report herein the synthesis and conformational analysis of Aβ_1–42_-amyloid related β-hairpin peptidomimetics, built on a piperidine–pyrrolidine semi rigid β-turn inducer and bearing two small recognition peptide sequences, designed on oligomeric and fibril structures of Aβ_1–42_. According to these peptide sequences, a stable β-hairpin or a dynamic equilibrium between two possible architectures was observed. These original constructs are able to greatly delay the kinetics of Aβ_1–42_ aggregation process as demonstrated by thioflavin-T fluorescence, and transmission electron microscopy. Capillary electrophoresis indicates their ability to preserve the monomer species, inhibiting the formation of toxic oligomers. Furthermore, compounds protect against toxic effects of Aβ on neuroblastoma cells even at substoichiometric concentrations. This study is the first example of acyclic small β-hairpin mimics possessing such a highly efficient anti-aggregation activity. The protective effect is more pronounced than that observed with molecules which have undergone clinical trials. The structural elements made in this study provide valuable insights in the understanding of the aggregation process and insights to explore the design of novel acyclic β-hairpin targeting other types of amyloid-forming proteins.

## Abstract 2

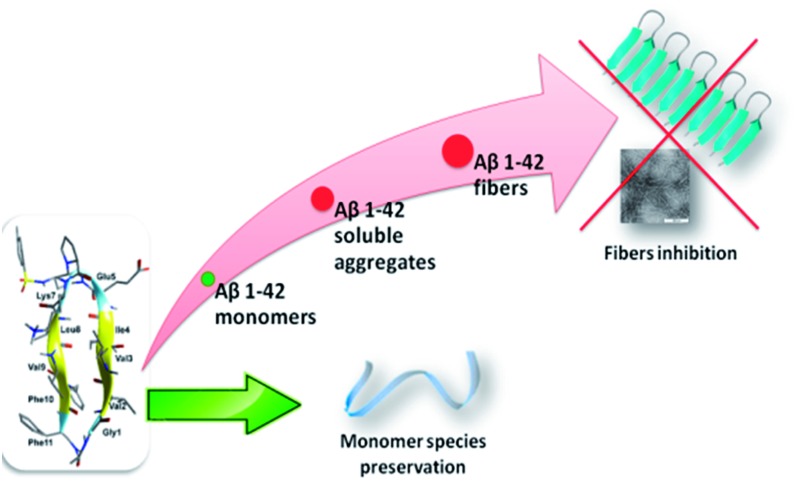
Acyclic β-hairpins designed on oligomeric and fibril structures of Aβ_1–42_ disrupt protein–protein interactions mediating amyloid β-peptide aggregation.

## Introduction

Amyloid fibrils are self-assembled insoluble aggregates characterized by highly ordered cross-β structures. They constitute the hallmark of more than 20 serious human amyloidosis diseases, such as Alzheimer's disease (AD), Parkinson's neurodegeneration, type II diabetes and spongiform encephalopathy.^1^ In particular, AD is associated with the aggregation of the amyloid-β (Aβ_1–42_) peptide into senile plaques in the brain.^2^ A large number of small molecules have been proposed for their ability to inhibit or modulate Aβ_1–42_ aggregation and toxicity. However, the aggregation process is highly complex, and extremely difficult to control.^3^ Fibrils are able to generate damaging redox activity and promote the nucleation of toxic oligomers.^4^ Recent studies indicate that soluble transient oligomers preceding fibril formation are highly toxic species.^5^ Their characterization and the activity of Aβ_1–42_ aggregation inhibitors on these small and toxic oligomeric species is generally lacking. Thus, the development of inhibitors targeting both oligomerization and fibrillization remains challenging despite its therapeutic significance.^4*c*^

Peptides are today reasonable alternatives to small molecule pharmaceuticals. They often offer greater efficacy, selectivity, specificity and a reduced risk of unforeseen side-reactions compared to small organic molecules, while some of their pharmacodynamic weaknesses can be circumvented by innovative formulations.^6^ A variety of small peptides that inhibit aggregation of Aβ and reduce its toxic effects have been already described.^7^ In particular, inhibition of Aβ-aggregation has been targeted using self-recognition elements (SREs). Indeed, molecules based on fragments of the Aβ-peptide, essentially on the nucleation sequence Aβ_16–20_ (KLVFF), were found promising as SREs.^8^ The design of macrocycles β-sheet mimics containing an unnatural tripeptide unit (Nowick's Hao) and SREs, has been a valid strategy.^9^ To our knowledge, the use of small acyclic β-hairpins has been very rarely explored as β-sheet binders and inhibitors of aggregation.^10^

**Fig. 1:**
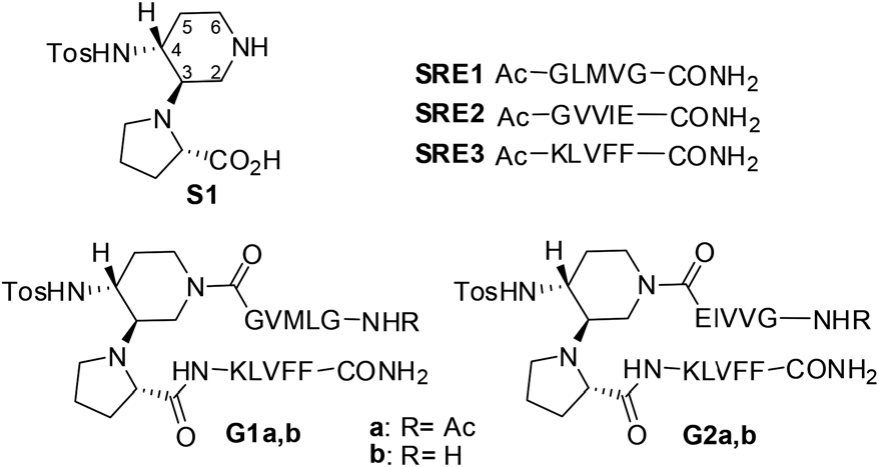
Structure of β-amyloid mimics **G1** and **G2** and the corresponding SREs.

Interestingly, compounds possessing several kinetically and thermodynamically accessible local minima representing conformations might be much more powerful inhibitors with respect to rigid ones in modulating protein–protein interactions.^11^ As Aβ-aggregation is a dynamic and complex process, we hypothesized that flexible β-hairpins could adapt themselves in the interaction with the different Aβ_1–42_ conformations present during the aggregation process, and in particular in the early stages of oligomerization. For that purpose, we designed two acyclic, β-hairpin mimics **G1** and **G2** based on the piperidine–pyrrolidine semi-rigid scaffold **S1**,^12^ developed recently as a flexible β-turn inducer (Fig. 1), and on different SREs of Aβ_1–42_. The nucleation sequence Aβ_16–20_ (KLVFF) has been introduced in the C-terminal sequence of both **G1** and **G2**. However, the choice of the N-terminal sequence was driven by the strategy to develop both a flexible and a more structured β-hairpin. The hydrophobic sequence G_33_LMVG_37_, facing K_16_LVFF_20_ in the more flexible oligomeric structures^13^ has been introduced in **G1**. In **G2**, GVVIE has been chosen as a mimic of the hydrophobic sequence G_38_VVIA_42_, facing K_16_LVFF_20_ in the stable fibril structures.^14^ The alanine residue has been replaced by glutamic acid in order to possibly engage an ionic interaction with the facing lysine residue, thus stabilizing the β-hairpin structure (Fig. 1). The N-terminal amino acid of both **G1** and **G2** was either acetylated (**G1a**, **G2a**) or not (**G1b**, **G2b**), in order to evaluate the capacity of the compounds to engage electrostatic interactions with acidic residues of Aβ_1–42_ and with the view to increase their affinity. Several computational and experimental studies on Aβ_1–42_ proved in fact that, in addition to the hydrophobic interactions involving in particular the 16–21 sequence (KLVFFA), the formation of a salt-bridge between amino acids Asp23 and Lys28 of amyloid might stabilize a turn motif involving residues 24–28.^13^ An interaction with Glu22 might be also promoted and beneficial for the activity of the molecules.^15^

## Results and discussion

### Conformational studies and synthesis

**Fig. 2:**
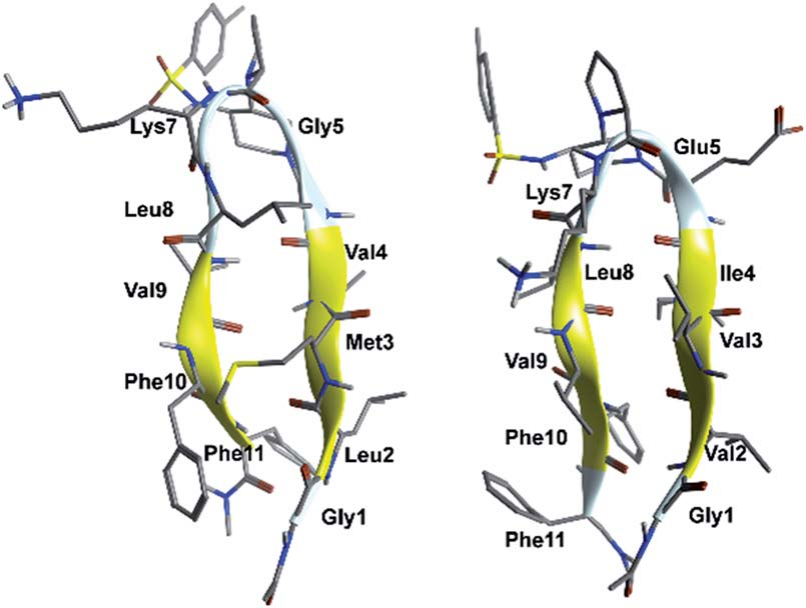
Representative structures of the most populated cluster obtained from cluster analyses of the 302.76 K trajectory of REMD simulations for peptides **G1a** (left) and **G2a** (right).

In order to evaluate the folding propensity of the designed **G1** and **G2** β-hairpin mimics, as well as to get preliminary information on their conformational stability, we performed a computational study using replica exchange molecular dynamics (REMD) on **G1a** and **G2a**.^16–18^ Thus, we simulated peptides **G1a** and **G2a** using the *ff96* force field coupled with the OBC(II) solvent model,^19^ (see ESI† for additional details). The secondary structure analysis by DSSP^20^ (Tables S1 and S2, ESI†) showed that both peptides have a relatively high tendency to form anti-parallel β-sheets. **G2a** seemed to form a very stable β-hairpin, with percentage values of anti-parallel β-sheet content, relatively to non-terminal amino acids, ranging from about 60 to about 90%. **G1a** was somehow less stable, with an anti-parallel β-sheet content averagely 20% less than **G2a**. In the H-bond analyses (Tables S3 and S4, ESI†) two pairs of very stable H-bonds, involving the backbone NH and C=O atoms of residues Ile4/Leu8 and Val2/Phe10, were observed for **G2a**. On the other hand, the occupancies of intramolecular H-bonds detected for **G1a** were lower. We observed a minor populated hairpin conformation, characterized by the H-bonds involving Val4/Leu8 and Leu2/Phe10, and a major “mismatched” hairpin involving Val4/Val9 and Leu2/Phe11. The representative structures of the most populated cluster for **G1a** and **G2a** (Fig. 2) showed a mismatched β-hairpin for the former peptide, with the N-terminal strand (Gly1–Gly5) that was shifted one residue with respect to the C-terminal strand (Lys7–Phe11). Conversely, for **G2a**, the two strands were perfectly matched. The higher conformational flexibility of **G1a**, compared to **G2a**, was also shown by the root mean square deviation (RMSD) analysis of the corresponding REMD trajectories (Fig. S1, ESI†), confirming the possibility of an equilibrium for the former peptide between multiple β-hairpin like conformations, while a single and fairly rigid β-hairpin conformation was predicted for **G2a**.

**Fig. 3:**
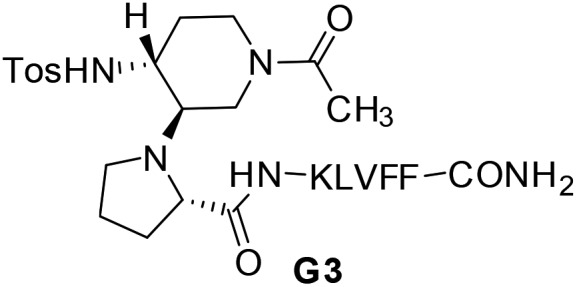
Structure of truncated mimic **G3**.

Compounds **G1** and **G2** were thus prepared by solid phase peptide synthesis, using the Fmoc strategy (see ESI† for details).^21^ In order to evaluate the efficacy of **G1** and **G2** molecules with respect to a truncated derivative or the single arms, we also prepared derivative **G3** (Fig. 3), containing the scaffold and only the Aβ (16–20) SRE, and compounds **SRE1–3** corresponding to the different SREs (Fig. 1, see ESI† for details).

**Fig. 4:**
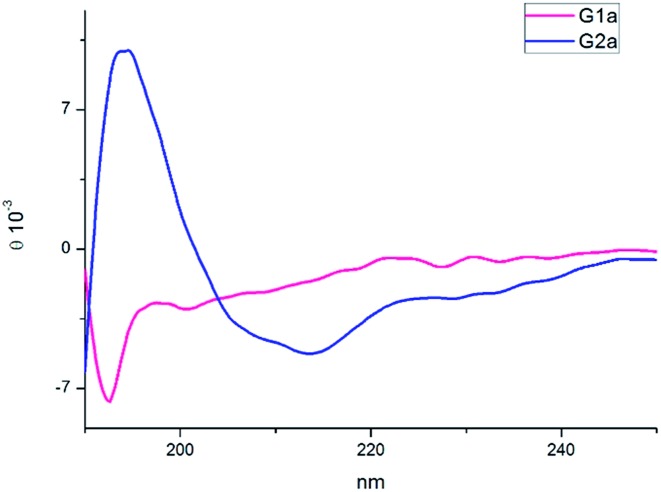
CD spectra of compounds **G1a** and **G2a** in MeOH.

The CD spectra of **G1a** and **G2a** were recorded in MeOH at 25 °C (Fig. 4). **G1a** showed a negative band at 195 nm indicating that in solution this peptidomimetic did not assume a preferred, single conformation. On the other hand, the spectrum of **G2a** was characterized by a strong positive Cotton effect at around 195 nm (π–π* energetic transition), and a negative band at around 215 nm (n–π* energetic transition), typical of β-sheet structures.

**Fig. 5:**
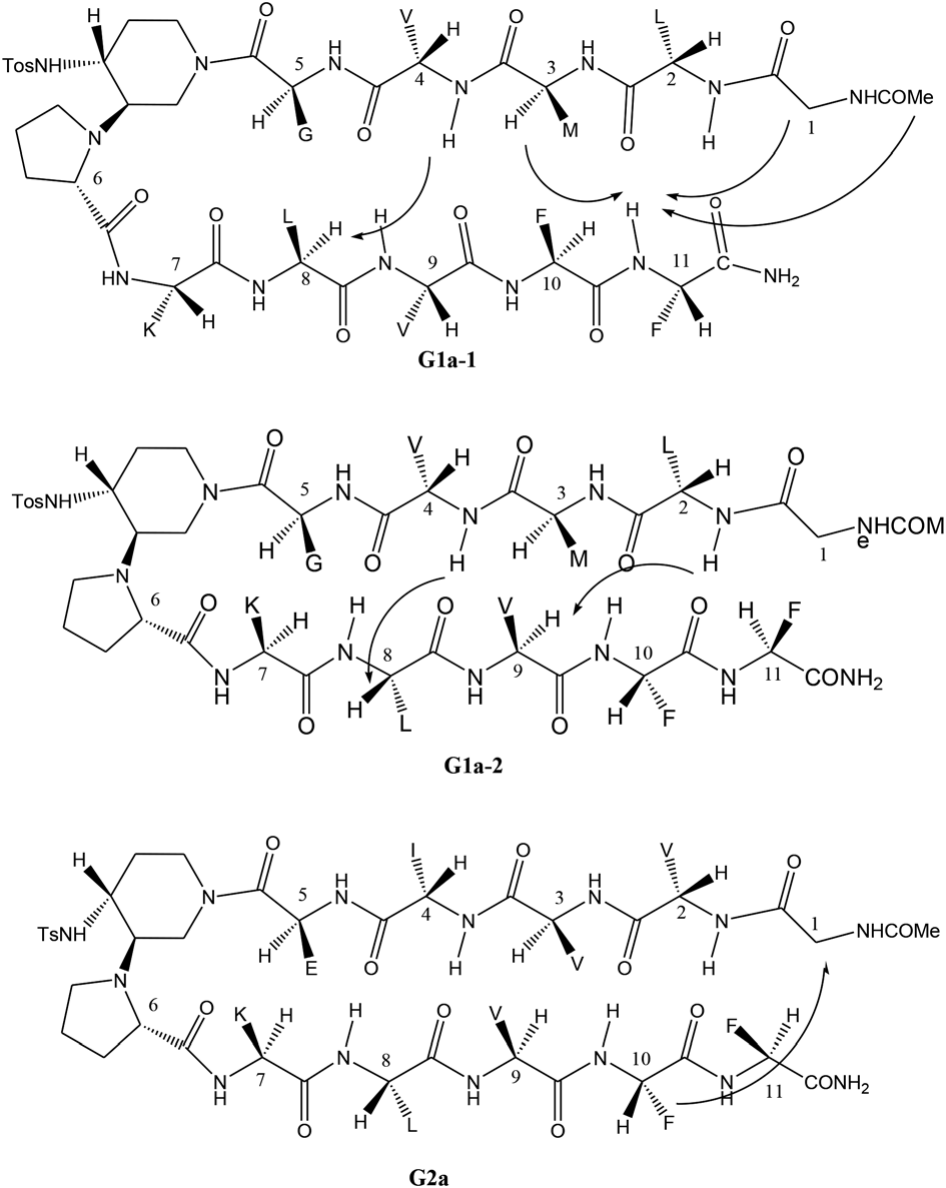
β-Hairpin structures of compounds **G1a-1**, **G1a-2**, and **G2a**, showing the assigned ROEs.

The different behaviour of **G1a** and **G2a** was confirmed by ^1^H-NMR experiments in CD_3_OH (Tables S6–S8 in ESI†). Compound **G1a** is present in solution as two different β-hairpin structures (**G1a-1**/**G1a-2**, 2 : 1 ratio, Fig. 5), characterized by a different alignment of the two peptide arms. This dynamic equilibrium is proved by the presence of several negative NH/NH ROEs (Fig. S4, ESI†).^22^ On the other hand, ^1^H NMR spectrum of **G2a** showed a good dispersion of the NH chemical shifts indicating the presence of a stable single β-hairpin conformation characterized by a peptide arms alignment similar to **G1a-2** (Fig. 5 on the bottom).^23^

ROESY experiments confirmed the presence of a turn structure in **G1a-1**, **G1a-2**, and **G2a**, as already reported for model sequences (Fig. S5 and S11, ESI†).^12*a*^

Several sequential CHα/NH ROEs, indicating β-conformations, were found for both **G1a-1** and **G1a-2** isomers (Fig. 5 and S6, ESI†). The different alignment of the peptide chains was proven by a ROE between NH_Phe11_/CHα_Met3_ in **G1a-1**, and by another one between NH_Leu2_/CHα_Val9_ in **G1a-2** (for a complete discussion see ESI†).

Regarding compound **G2a** we could detect only one β-hairpin diagnostic ROE between CHα_Gly1_ and the phenyl ring of Phe-10 (Fig. 5 and S12, ESI†). Several CHα signals are indeed overlapped or masked by the solvent. The presence of a β-hairpin structure was confirmed by ^3^ *J*HN/CHα coupling constants that are higher than 8 Hz (Table S9, ESI†).^24,25^

**Fig. 6:**
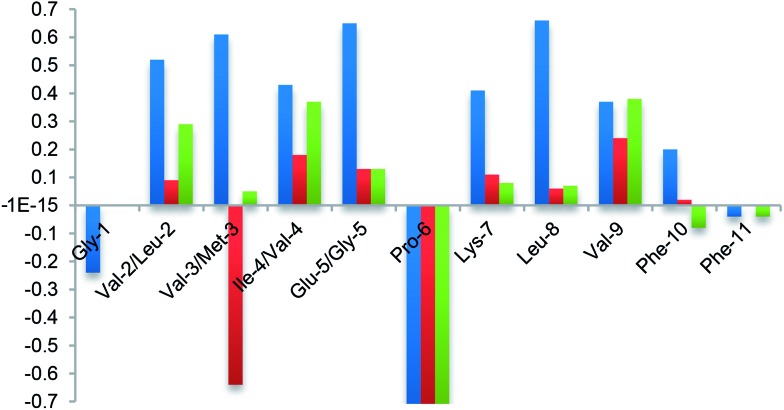
NMR analysis. Plot of difference between Hα chemical shift values in the random coil and the values determined experimentally for **G2a** (blue) and isomers **G1a-1** (red) and **G1a-2** (green) in CD_3_OH at 298 K.

Finally, the β-hairpin conformation was definitively confirmed for all compounds by the positive difference between experimental Hα chemical shift values and “random” ones^26^ (Fig. 6). Only Met-3 of **G1a-1** is characterized by a negative Δ*δ*αH value. This is probably due to the anisotropic effect^27^ of the aromatic ring of Phe-11 that faces Met-3, as evicted from ROESY experiments (Fig. S6A, ESI†).

Taking together both experimental and theoretical results, we can conclude that different hairpin architectures are possible for **G1a** and **G2a**, depending on the N-terminus sequence. The GVVIE motif in **G2a** strongly stabilizes a single “matched” hairpin conformation. On the other hand, the GLMVG motif in **G1a** gave a dynamic equilibrium between two possible architectures, the “mismatched” hairpin being the more stable.

### Inhibition of Aβ_1–42_ fibrillization

**Fig. 7:**
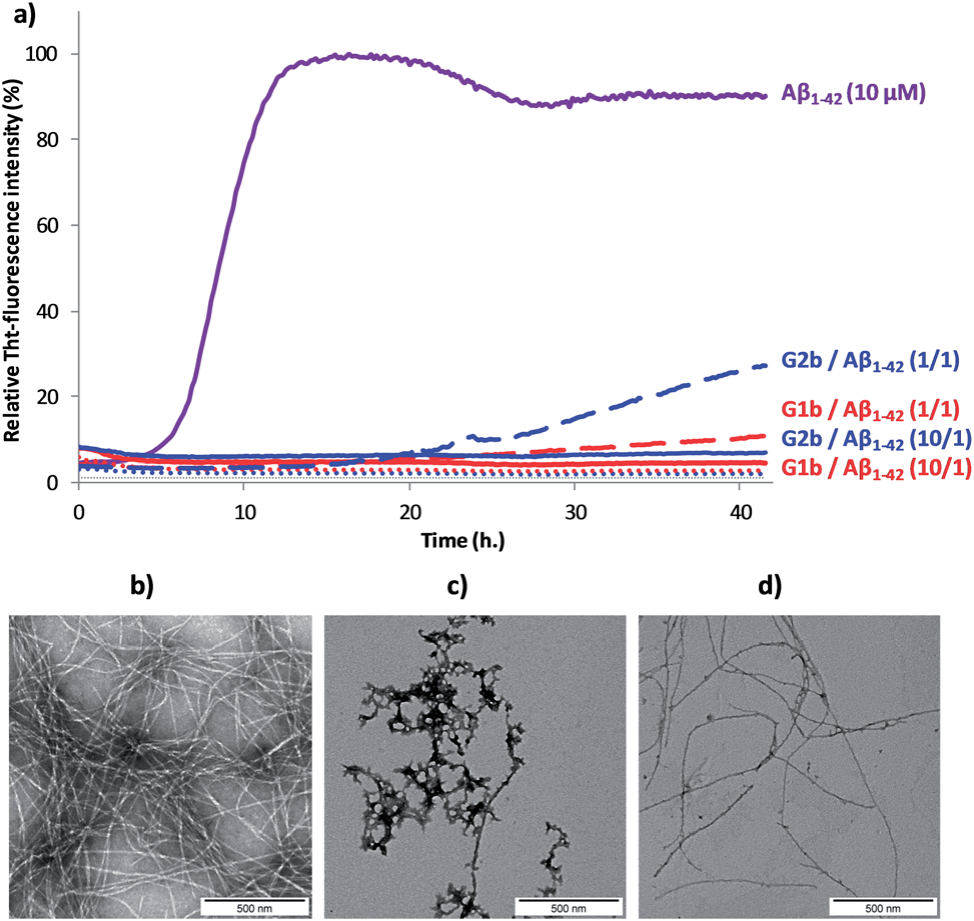
(a) Representative curves of ThT fluorescence assays over time showing Aβ_1–42_ (10 μM) aggregation in the absence (purple curve) and in the presence of compounds **G1b** (red curves) and **G2b** (blue curves) at compound/Aβ_1–42_ ratios of 10/1 and 1/1. The control curves are represented in dotted lines (**G1b** in red, **G2b** in blue and grey for buffer). Fibril formation of Aβ_1–42_ visualized by TEM: negatively stained images recorded after 42 h of incubation of Aβ_1–42_ (10 μM in 10 mM Tris·HCl, 100 mM NaCl at pH = 7.4) alone (b) or in the presence of 10 μM of **G1b** (c) or **G2b** (d). Scale bars represent 500 nm.

The ability of compounds **G1–3** and **SRE1–3** to interact with Aβ_1–42_ during the fibrillization process was first studied by thioflavin-T (ThT) fluorescence spectroscopy.^28^ The fluorescence curve for Aβ_1–42_ at a concentration of 10 μM followed the typical sigmoid pattern with a lag phase of 4–5 h followed by an elongation phase and a final plateau reached after 10–12 h (Fig. 7a). Two parameters were derived from the ThT curves of Aβ_1–42_ alone and in the presence of the evaluated compound: (1) *t*_1/2_, is defined as the time at which the half maximal ThT fluorescence is observed, which gives insight on the rate of the aggregation process; (2) the fluorescence intensity at the plateau (*F*) which is assumed to depend on the amount of fibrillar material formed (Table 1).

**Table 1:** Effects of compounds **G1–2** on Aβ_1–42_ fibrillization assessed by ThT-fluorescence spectroscopy at 10/1 and 1/1 compound/Aβ ratios (the concentration of Aβ_1–42_ is 10 μM) and compared to the values obtained for Aβ_1–42_ alone (*t*_1/2_ and *F*)^*a*^

Compounds (Compound/Aβ ratio)	*t*_1/2_ extension^*b*^	Change of fluorescence intensity at the plateau^*c*^ (%)
**G1a** (10/1)	NA	–97 ± 1%
**G1a** (1/1)	2.06 ± 0.12	–71 ± 2%
**G2a** (10/1)	Sat^*d*^	Sat^*d*^
**G2a** (1/1)	1.76 ± 0.11	–41 ± 7%
**G1b** (10/1)	NA	–97 ± 1%
**G1b** (1/1)	NA	–90 ± 2%
**G2b** (10/1)	NA	–95 ± 1%
**G2b** (1/1)	>3.56 ± 0.12	–73 ± 3%

^*a*^NA = no aggregation, parameters are expressed as mean ± SE, *n* = 3–6.

^*b*^See ESI for the calculation of the *t*_1/2_ extension. A compound displaying a *t*_1/2_ increase >1 is a delayer of aggregation.

^*c*^See ESI for the calculation of the change of fluorescence intensity at the plateau.

^*d*^Sat means that a saturation of the fluorescence signal is observed because **G2a** self-aggregates at 100 μM.

Both **G1** and **G2** series are able to inhibit Aβ_1–42_ aggregation. The **G1** series, containing the sequence G_37_VMLG_33_, and possessing a dynamic equilibrium between two different β-hairpin conformations, exerts a slightly superior inhibitory activity (Fig. 7 and Table 1). Furthermore, the free terminal amine is also important for Aβ_1–42_ aggregation suppression. Unprotected **G1b** and **G2b** were indeed able to totally suppress aggregation at compound/Aβ_1–42_ ratio of 10/1 and still dramatically delayed Aβ_1–42_ aggregation at 1/1 ratio (Fig. 7a and Table 1). Acetylated derivatives **G1a** and **G2a** retained this activity, but to a lesser extent (Table 1 and Fig. S14†). This result supports our hypothesis on the importance of establishing an ionic interaction between the N-terminal amino group and acidic residues of Aβ_1–42_.

No activity was observed for the isolated pentapeptides GLMVG (**SRE1**) and GVVIE (**SRE2**) (Table S11 and Fig. S14†). KLVFF (**SRE3**) delayed Aβ_1–42_ aggregation at compound/Aβ_1–42_ ratio of 10/1,^8a,29^ however in a much lesser extent than **G1** and **G2** series, while exerted no activity at 1/1 ratio (Table S11†). The **G3** intermediate containing KLVFF linked to the piperidine–pyrrolidine scaffold **S1** is more active than **SRE3**. These results highlight that the piperidine-pyrrolidine scaffold **S1** and the pentapeptide KLVFF are both crucial for the activity, but the whole β-hairpin construct is necessary to strongly delay the Aβ_1–42_ aggregation kinetics.

In order to assess the selectivity on Aβ_1–42_ peptide, the ability of compounds **G1b** and **G2b** to interact with IAPP (islet amyloid polypeptide), an amyloid protein involved in type 2 diabetes mellitus but having another SRE,^30^ was also tested by the ThT-fluorescence assay under conditions similar to that described for Aβ_1–42_ peptide. It is noteworthy that both compounds displayed no activity on IAPP fibrillization process at compound/Aβ_1–42_ ratio of 1/1 and only slightly delayed it at the higher ratio (10/1) (Fig. S15†). This result suggests that the inhibition of aggregation displayed by compounds **G1b** and **G2b** on Aβ_1–42_ peptide is sequence specific.

Transmission electron microscopy (TEM) analyses were performed on the most promising **G1a**, **G1b** and **G2b** compounds. Images were recorded at 20 h and 42 h of fibrillization kinetics with samples containing 10 μM of each compound corresponding to the compound/Aβ_1–42_ ratio of 1/1 (Fig. 7b–d and S16†). Differences were observed in both quantity and morphology of aggregates formed. At 42 h, a very dense network of fibers displaying a typical morphology was observed for Aβ_1–42_ alone (Fig. 7b). In the samples containing **G1a**, the network of fibers was significantly less dense than in the control experiment after 20 h and 42 h. However, the fibers displayed the same morphology (Fig. S16, ESI†). In the samples containing **G2b**, the same trends as with **G1a** were observed (Fig. 7d and S16†). In samples containing **G1b**, we mainly observed globular aggregates after 20 h and 42 h (Fig. 7c and S16) indicating that the aggregation pathway could be different from the one observed for Aβ_1–42_ alone. These results validated the ThT-fluorescence data, indicating that compounds **G1a**, **G1b** and **G2b** dramatically slowed down the aggregation of Aβ_1–42_ and efficiently reduced the amount of typical amyloid fibrils.

### Inhibition of Aβ_1–42_ oligomerization

**Fig. 8:**
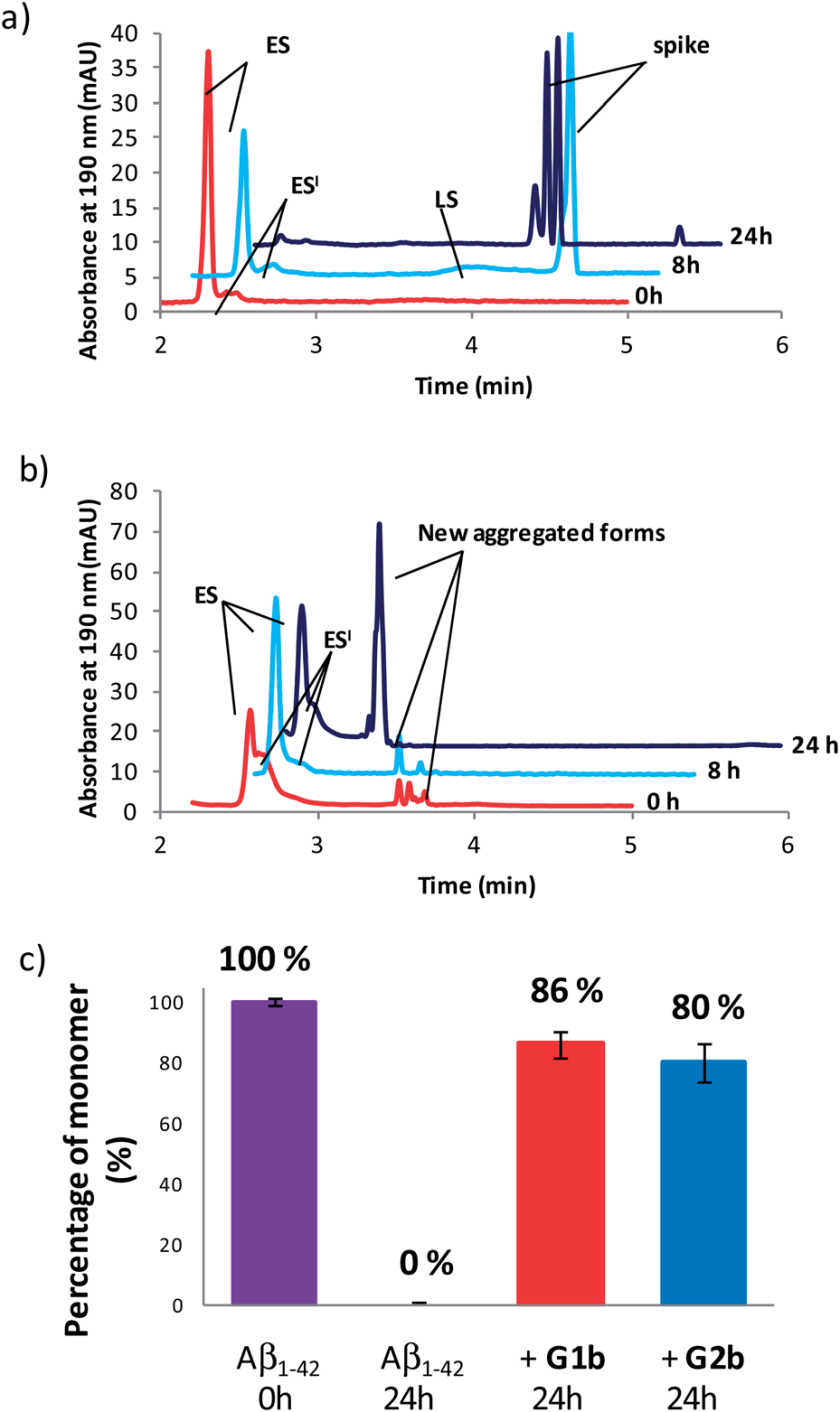
Electrophoretic profile obtained immediately (0 h, red), 8 h (blue) and 24 h (purple) after sample dissolution of Aβ_1–42_ peptide (100 μM) (a) alone or (b) in the presence of **G1b** (100 μM). (c) Peak area of the monomer (ES) related to its peak area in the sample of Aβ_1–42_ alone at 0 h.

Compounds **G1b** and **G2b** were finally studied (at compound/Aβ_1–42_ ratio of 1/1) by Capillary Electrophoresis (CE) using a method we recently proposed to monitor the very early steps of the oligomerization process overtime and to analyze the effect of drugs on these challenging first stages.^31^ We focused our attention on three kinds of species: (1) the monomer (peak ES), (2) different small metastable oligomers grouped under peak ES′ and (3) transient species formed later and which correspond to species larger than dodecamers but still soluble (peak LS). Aggregation kinetics of Aβ_1–42_ peptide alone (Fig. 8a and S18†) showed that overtime, the monomer ES peak decreased in favor of the oligomer peaks ES′ and LS, and that insoluble species, forming spikes in the profile, appeared after 8 hours.

In the presence of **G1b**, the aggregation kinetics of Aβ_1–42_ peptide was greatly modified (Fig. 8b and S19†). Noteworthy, the monomeric species (peak ES) was dramatically stabilized. 86% of the monomer remained after 24 h in the presence of **G1b**, while it was no more detected in the control sample (Fig. 8c). Moreover, the larger aggregated species LS (>dodecamers) were not detected. New aggregated forms of Aβ_1–42_, between ES′ and LS migration times were observed on each electrophoretic profile. We checked that these new aggregated forms were not due to **G1b** degradation or self-assemblies (Fig. S17A†). They were probably aggregated forms with a different morphology than both LS and those giving spikes observed in Aβ_1–42_ control. This observation is in accordance with the TEM images where globular aggregates were observed instead of the classical dense network of fibers (Fig. 7c and S16†). In ThT-assays, no fluorescence was detected, indicating that the globular species were not characterized by highly ordered β-structures (Fig. 7a). Remarkably, the presence of the monomer was maintained even after 4 days (Fig. S19B†). We concluded that **G1b** is able to prevent the formation of toxic soluble oligomers of Aβ_1–42_ peptide and to maintain the presence of the non toxic monomer overtime.

**G2b** also dramatically maintained the presence of the monomer (peak ES, 80% after 24 h, Fig. 8c, S20 and S21†). However, new aggregated forms were only transiently observed but were not anymore detected after 24 h. This result was also in accordance with the TEM images where we observed a much less dense network of fibers, although the typical morphology was retained.

### Protection against Aβ_1–42_ cell toxicity

**Fig. 9:**
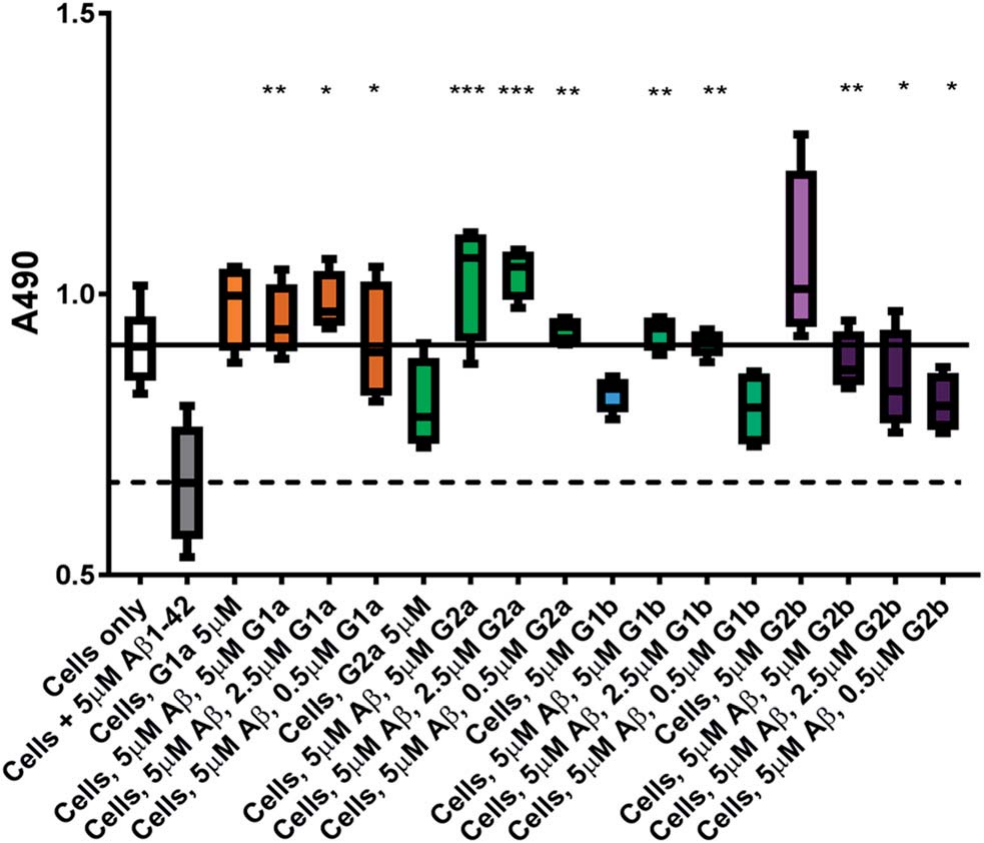
Cell viability assay results. The solid line represents the absorbance value seen for cells incubated without Aβ_1–42_ (white box) and the dotted line that seen for cells incubated with 5 μM Aβ_1–42_ (grey box). A statistically significant difference between Aβ_1–42_ treated cells with and without inhibitor is indicated by */**/*** corresponding to *p* > 0.05/0.01/0.001. *n* = 4 for each condition.

**Fig. 10:**
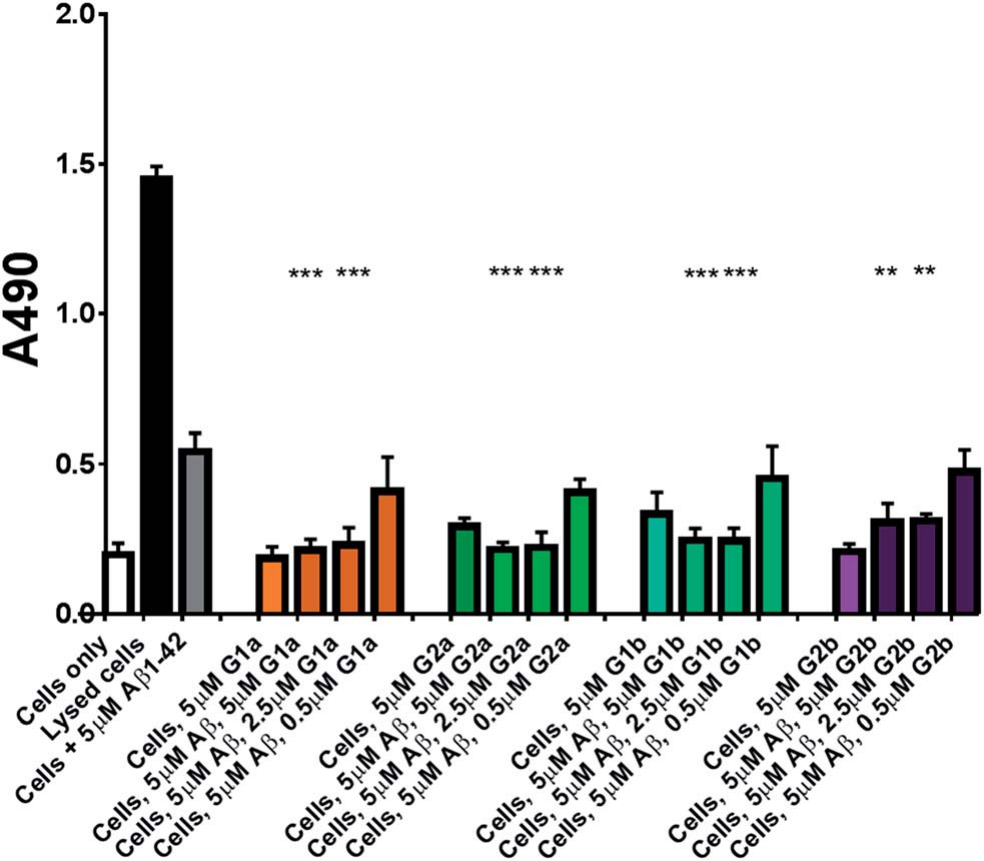
LDH based cell toxicity test. Cells were treated in the same manner as with the MTS assay, and cell proliferation was measured using the CytoTox 96® NonRadioactive Cytotoxicity Assay Protocol from Promega. Statistical analysis was performed using a Student's test comparing the results for cells exposed to 5 μM Aβ_1–42_ with and without inhibitor where ** = *p* < 0.01 and *** = *p* < 0.001.

The inhibitors were investigated to determine their ability to reduce the toxicity of aggregated Aβ_1–42_ to SH-SY5Y neuroblastoma cells. The addition of all compounds, to a lesser extent for **G2b**, showed a protective effect on cell survival (MTS assay, Fig. 9) and membrane damage (LDH membrane integrity assay, Fig. 10) in the presence of cytotoxic 5 μM Aβ_1–42_. Remarkably, this protective effect was seen at equimolar amounts of inhibitor to Aβ_1–42_ and was still significant at a very low ratio of 0.1/1 (inhibitor/Aβ_1–42_) in the MTS assay. Both **G2a** and **G1b** showed a slight negative effect on cell viability when incubated with cells alone, although this was negated when Aβ was present.

**Fig. 11:**
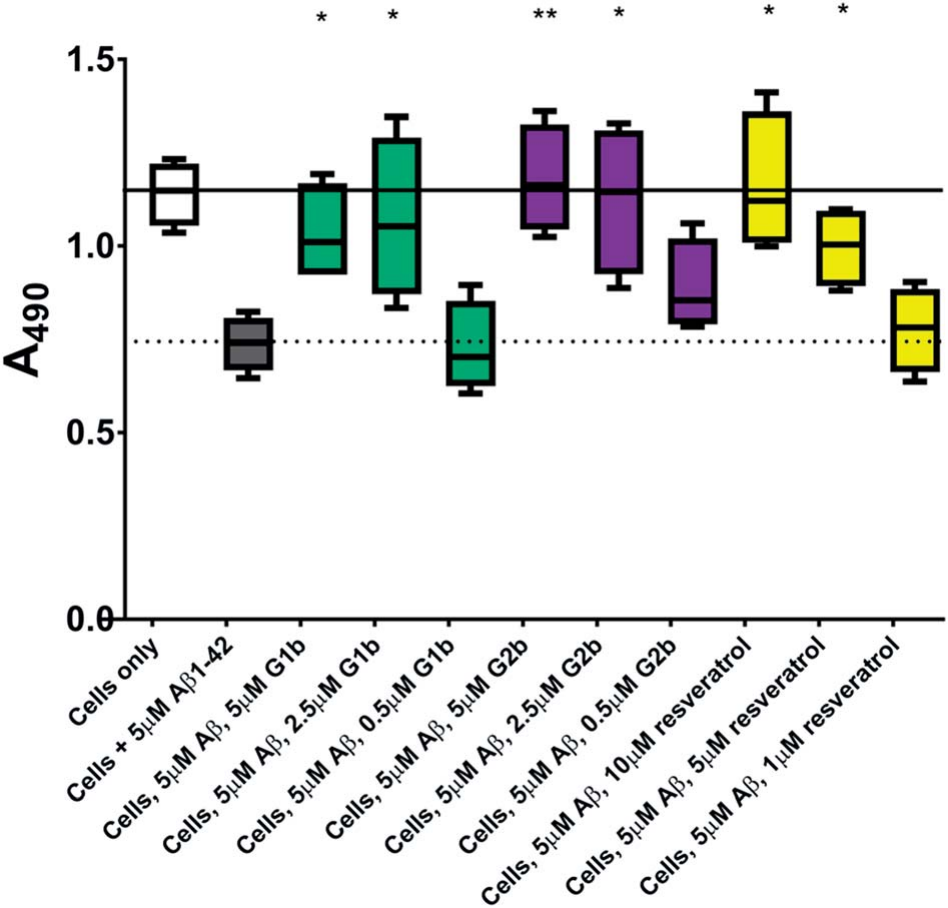
Cell viability assay results of resveratrol compared to **G1b** and **G2b**. The solid line represents the mean absorbance value seen for cells incubated without Aβ_1–42_ (white box) and the dotted line that seen for cells incubated with 5 μM Aβ_1–42_ (grey box). A statistically significant difference between Aβ_1–42_ treated cells with and without inhibitor is indicated by */**/*** corresponding to *p* > 0.05/0.01/0.001. *n* = 4 for each condition.

This protective effect is more marked than that observed with molecules which have undergone clinical trials^32–34^ or other molecules recently described as efficient reducers of Aβ_1–42_ toxicity.^35^ In particular, in the literature, resveratrol was reported to protect SH-SY5Y neuroblastoma cells from Aβ_1–42_ toxicity at 10/1 and 2/1 (resveratrol/Aβ_1–42_) ratios,^32^ scyllo-inositol was demonstrated to protect PC-12 cells at 10/1 ratio (scyllo-inositol/Aβ_1–42_),^33^ and (–)-epigallocatechin-3-gallate (EGCG) protected murine neuro-2a neuroblastoma cells at 1/1 ratio (–)-epigallocatechin-3-gallate/Aβ_1–42_.^34^ In our hands, and comparable to the published data,^32^ resveratrol efficiently protected SH-SY5Y neuroblastoma cells only at a ratio of 2/1 (resveratrol/Aβ_1–42_). A stoichiometric ratio 1/1 was less efficient than a substoichiometric ratio of **G1b** and **G2b** (0.5/1 compound/Aβ_1–42_) (Fig. 11). Resveratrol exhibits multi-target activity and thus is not selective for Aβ_1–42_ aggregation. For example, resveratrol inhibits similarly the aggregation of other amyloid proteins such as IAPP^36^ (EGCG also inhibits similarly Aβ_1–42_ and IAPP aggregation in ThT fluorescence assays^37,38^), which is not the case for **G1b** and **G2b**, as mentioned above. By choosing the SREs in our β-hairpin mimics, specifically according to the target amyloid proteins, we can modulate the activity and expect selective activities.

## Conclusion

We described new β-hairpin mimics designed on oligomeric and fibril structures of Aβ_1–42_ and containing a piperidine–pyrrolidine β-turn inducer. The presence of two small recognition sequences able to engage both hydrophobic and ionic interactions with Aβ_1–42_, dramatically increased the inhibitory effect on the fibrillization process. Furthermore, the presence of the semi-rigid piperidine–pyrrolidine scaffold **S1** and of the hydrophobic sequence G_33_LMVG_37_, which allows a dynamic equilibrium between different architectures, leads to the obtainment of compound **G1b** able to inhibit totally the formation of amyloid fibrils. As far as we know, this study is the first example of acyclic small β-hairpin mimics possessing such a highly efficient anti-aggregation activity. This activity is much higher than isolated SREs described in the literature. Furthermore, to the best of our knowledge, this is the first example of compounds able to dramatically preserve the non toxic monomer species of Aβ_1–42_. This result might explain the mechanism by which β-hairpin mimics exhibit a strong protective effect on cells even at substoichiometric concentrations. The structural elements made in this study provide valuable insights to explore the design of novel acyclic β-hairpin targeting other types of amyloid-forming proteins.
