# Supplementary material for: β-Hairpin mimics containing a piperidine–pyrrolidine scaffold modulate the β-amyloid aggregation process preserving the monomer species
Source: Chem Sci. 2016 Oct 7;8(2):1295–302. doi: 10.1039/c6sc03176e (PMC5359901; doi:10.1039/c6sc03176e)
Supplement: Supplementary file 1 [file SC-008-C6SC03176E-s001.pdf]

Electronic Supplementary Material (ESI) for Chemical Science.
This journal is © The Royal Society of Chemistry 2016

Supporting information

β-Hairpin mimics containing a piperidine-pyrrolidine scaffold modulate the β-amyloid aggregation process preserving the monomer species

Sara Pellegrino[a]*, Nicolò Tonali[b], Emanuela Erba[a], Julia Kaffy[b], Myriam Taverna[c], Alessandro Contini[a], Mark Taylor[d], David Allsop[d], Maria Luisa Gelmi[a], Sandrine Ongeri[b]

[a] DISFARM-Sez. Chimica Generale e Organica "A.Marchesini", Università degli Studi di Milano, via Venezian 21, 20133 Milano, Italy

[b] Molécules Fluorées et Chimie Médicinale, BioCIS, Univ. Paris-Sud, CNRS, Université Paris Saclay, 5 rue Jean-Baptiste Clément, 92296 Châtenay-Malabry Cedex, France.

[c] Protéines et Nanotechnologies en Sciences Séparatives, Institut Galien Paris-Sud, Univ. Paris-Sud, CNRS, Université Paris Saclay, 5 rue Jean-Baptiste Clément, 92296 Châtenay-Malabry Cedex, France.

[d] Lancaster University, Division of Biomedical and Life Sciences, Faculty of Health and Medicine, Lancaster LA1 4YQ, UK

Table of contents

Page S3 Computational Methods

Page S4 Computational Additional Figures and Tables

Page S7 Experimental Materials and Methods

Page S7 Synthesis of Fmoc Protected Scaffold **4**

Page S9 Synthesis of Compounds **SRE1-3**, **G1-3**

Page S12 Circular Dichroism of compounds **G1a** and **G2a**

Page S13 NMR discussion for compounds **G1a** and **G2a**

Page S14 NMR data for compounds **G1a** and **G2a**

Page S27 Experimental procedure for fluorescence-detected thioflavin-T binding assay (Aβ_{1-42}).

Page S28 Representative curves of Tht fluorescence assays over time showing Aβ_{1-42} aggregation in presence of compounds **G1a**, **G2a**, **G3**, **SRE1**, **SRE2**, **SRE3**

Page S29 Experimental procedure for fluorescence-detected thioflavin-T binding assay (IAPP)

Page S30 Representative curves of Tht fluorescence assays over time showing IAPP aggregation in presence of compounds **G1b** and **G2b**

Page S31 Experimental procedure for transmission electron microscopy studies, and images recorded for compounds **G1a**, **G1b** and **G2b**

Page S32 Capillary electrophoresis protocol

Page S35-S36 Cell toxicity procedure

Page S37 Bibliography

Computational Methods

The parameters for the piperidine-pyrrolidine semi-rigid scaffold were derived by following the same protocol previously reported for similar studies.[1,2] Briefly, the scaffold geometry was built using MOE,[3] capped with an acetyl (Ac) and a NHMe group at the N- and C-termini, respectively, and submitted to a conformational search using the "Low Mode" method implemented in MOE (MMFF94x force field, Born solvation, iteration limit = 40000, MM iteration limit = 2500, rejection limit = 500). The two lowest energy conformations, selected for partial charges parameterization with the R.E.D.IV,[4] were optimized at the HF/6-31G(d) level. Two different spatial orientations were used to derive RESP-A1 charges.

REMD simulations were performed on **G1a** and **G2a** peptides, built with the tLEaP module of AMBER 14 by imposing an extended conformation ($\varphi = \psi = \omega = 180°$).[5] For simulations with the AMBER ff96 force field and GB-OBC(II) solvent model (igb = 5, mbondi2 set of radii).[6,7] Structures were initially geometry-optimized by 500 steps of steepest-descendant followed by 500 steps of conjugated-gradient minimization. For the REMD simulation, 20 replicas were distributed over the following temperatures 260.00, 273.56, 287.80, 302.76, 318.46, 334.96, 352.29, 370.50, 389.61, 409.67, 430.72, 452.83, 476.02, 500.35, 525.91, 552.75, 580.88, 610.40, 641.39 and 673.90 K, accordingly to the T-REMD server.[8] A short equilibration run (200 ps) was performed on each replica prior to the actual production run. REMD simulations were conducted with pmemd on each peptide for 50 ns at constant temperature by using Langevin dynamics (ntt = 3) with different seeds (ig) for every simulation.[5] A time step of 0.002 ps and an infinite cut-off for electrostatic were requested, and the SHAKE algorithm was used in order to constrain all bonds involving hydrogens.[9] Exchanges were attempted every 2 ps and were on average accepted with a 55% probability. The trajectories at 302.76 K of the REMD simulations were extracted and analyzed. To evaluate convergence, H-bonds, DSSP,[10] cluster population, and geometry of cluster representative structure, were monitored every 5 ns, showing convergence after the first 25 ns, so all production analyses were performed on the 25 ns – 50 ns fraction of the 302.76 K REMD trajectory. H-bond analyses were performed by setting a donor-acceptor distance cutoff of 4.0 Å and a Donor-H-acceptor angle cutoff of 120 deg. Cluster analyses were performed with cpptraj using the average-linkage algorithm and the pairwise mass-weighted Root Mean Square Displacement (RMSD) on backbone heavy atoms as a metric. A total of 10 cluster were requested on the basis of the pseudo-F statistical analysis, and SSR/SST ratio (R-squared value).[11]

Computational Additional Figures and Tables

Table S1. Full DSSP Secondary Structure Analysis Results of the 302.76 K Trajectory of REMD Simulation for **G1a**.

	**secondary structure (%)**						
**#Residue**	**Para**	**Anti**	**3-10**	**Alpha**	**Pi**	**Turn**	**Bend**
Ace	0.00	0.00	0.00	0.00	0.00	0.00	0.00
Gly1	0.01	14.24	0.00	0.00	0.00	0.02	0.00
Leu2	0.03	48.17	0.00	0.00	0.00	0.04	0.00
Met3	0.00	73.46	0.00	0.00	0.00	0.03	1.03
Val4	0.06	63.33	0.00	0.00	0.00	3.50	12.40
Gly5	0.17	0.66	0.00	0.00	0.00	45.97	38.10
S1	0.00	0.00	0.00	0.00	0.00	47.17	52.66
Lys7	0.00	5.76	0.00	0.00	0.00	41.82	51.46
Leu8	0.06	22.94	0.00	0.00	0.00	24.60	10.12
Val9	0.14	62.81	0.00	0.00	0.00	0.60	1.27
Phe10	0.05	68.79	0.00	0.00	0.00	0.00	0.00
Phe11	0.00	39.51	0.00	0.00	0.00	0.00	0.00
NHMe	0.00	0.00	0.00	0.00	0.00	0.00	0.00

Table S2. Full DSSP Secondary Structure Analysis Results of the 302.76 K Trajectory of REMD Simulation for **G2a**.

	**secondary structure (%)**						
**#Residue**	**Para**	**Anti**	**3-10**	**Alpha**	**Pi**	**Turn**	**Bend**
Ace	0.00	0.00	0.00	0.00	0.00	0.00	0.00
Gly1	0.07	22.74	0.00	0.00	0.00	0.03	0.00
Val2	0.01	65.18	0.00	0.00	0.00	0.03	0.00
Val3	0.00	93.50	0.00	0.00	0.00	0.00	0.14
Ile4	0.01	82.98	0.00	0.00	0.00	0.00	2.95
Glu5	0.00	0.00	0.00	0.00	0.00	83.45	16.47
S1	0.00	0.00	0.00	0.00	0.00	83.45	16.55
Lys7	0.02	7.36	0.00	0.00	0.00	76.73	14.75
Leu8	0.00	83.04	0.00	0.00	0.00	1.08	0.24
Val9	0.00	93.28	0.00	0.00	0.00	0.02	0.14
Phe10	0.06	61.70	0.00	0.00	0.00	0.05	0.00
Phe11	0.02	18.96	0.00	0.00	0.00	0.04	0.00
NHMe	0.00	0.00	0.00	0.00	0.00	0.00	0.00

Table S3. Results of H-bond analysis of the 302.76 K REMD Trajectory of **G1a**

#Acceptor	Donor	Occ%	Avg Distance (Å)	Avg Angle (deg.)
VAL9 C=O	VAL4 N-H	23.5	3.1	160.8
VAL4 C=O	VAL9 N-H	23.2	3.1	161.7
LEU2 C=O	PHE11 N-H	20.9	3.2	154.7
LEU8 C=O	VAL4 N-H	15.2	3.1	161.9
LEU2 C=O	PHE10 N-H	14.7	3.1	156.4
VAL4 C=O	PHE10 N-H	13.4	3.1	160.7
PHE10 C=O	VAL4 N-H	13.4	3.1	161.0
VAL9 C=O	MET3 N-H	12.1	3.1	155.6
VAL4 C=O	LEU8 N-H	12.0	3.3	144.1
GLY1 C=O	PHE11 N-H	11.3	3.1	156.3
PHE11 C=O	LEU2 N-H	10.9	3.2	153.2
LEU2 C=O	NME N-H	10.3	3.2	156.1
PHE10 C=O	MET3 N-H	9.3	3.1	156.1
MET3 C=O	PHE10 N-H	8.7	3.1	158.2
LEU8 C=O	MET3 N-H	8.3	3.0	156.3
PHE10 C=O	LEU2 N-H	8.2	3.2	152.5
GLY1 C=O	PHE10 N-H	7.3	3.2	155.8
GLY1 C=O	NME N-H	7.3	3.2	155.9
MET3 C=O	LEU8 N-H	6.8	3.1	153.1
MET3 C=O	VAL9 N-H	6.8	3.4	153.9
LEU8 C=O	GLY5 N-H	6.5	3.2	154.2
S1 C=O	LEU8 N-H	6.5	3.5	135.1
PHE11 C=O	GLY1 N-H	6.0	3.3	151.3

Table S4. Results of H-bond analysis of the 302.76 K REMD Trajectory of **G2a**

#Acceptor	Donor	Occ%	Avg Distance (Å)	Avg Angle (deg.)
LEU8 C=O	ILE4 N-H	88.5	3.0	161.6
VAL2 C=O	PHE10 N-H	83.2	3.1	157.2
ILE4 C=O	LEU8 N-H	68.5	3.4	145.9
PHE10 C=O	VAL2 N-H	53.1	3.2	156.4
ACE C=O	NME N-H	15.9	3.3	153.3
C386 C=O	LEU8 N-H	9.9	3.7	133.7
LYS7 C=O	ILE4 N-H	6.7	3.1	160.0
ILE4 C=O	LYS7 N-H	6.6	3.3	159.2
VAL2 C=O	VAL9 N-H	6.6	3.2	161.0
VAL9 C=O	VAL2 N-H	6.3	3.1	157.9
ACE C=O	PHE11 N-H	3.7	3.3	155.8
PHE10 C=O	NME N-H	2.5	3.4	132.2
VAL2 C=O	LEU8 N-H	1.4	3.1	153.7
VAL2 C=O	PHE11 N-H	1.4	3.2	155.5
LEU8 C=O	VAL2 N-H	1.4	3.1	158.0

VAL9 C=O	ILE4 N-H	1.3	3.1	160.0
PHE10 C=O	GLY1 N-H	1.2	3.2	144.1
LYS7 C=O	VAL9 N-H	1.2	3.5	125.7
GLY1 C=O	VAL3 N-H	1.2	3.4	131.9
PHE11 C=O	VAL2 N-H	1.1	3.2	155.7
S1 C=O	ILE4 N-H	1.0	3.4	151.1

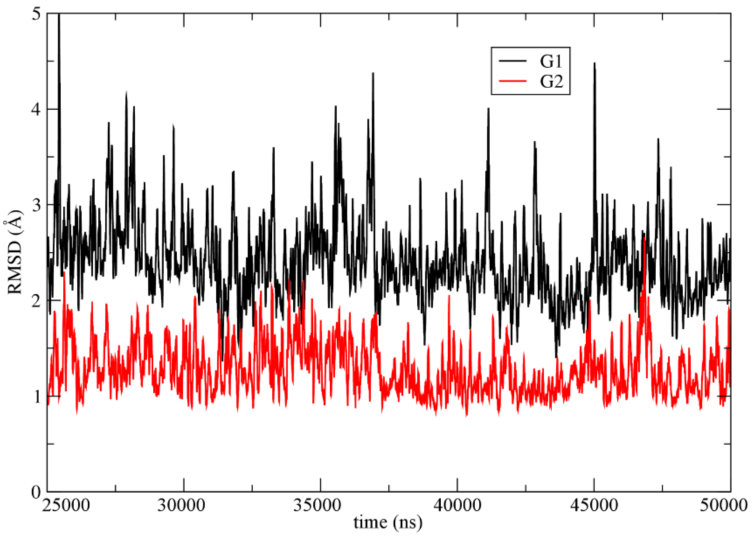

Figure S1. Comparison of the RMSD vs simulation time patterns obtained by RMSD analyses of the 302.76 K REMD trajectories (25 – 50 ns) of peptides **G1a** and **G2a**. The most representative structure obtained by cluster analysis was used as a reference (backbone heavy atoms).

Experimental Materials and Methods

Solvents, reagents were purchased from commercial sources. All peptides were purified using RP-HPLC and a C-18 column (10 µm, 250   22 mm). ESI mass spectra were recorded on a LCQ Advantage spectrometer.

Synthesis of Fmoc-protected scaffold 4

The Fmoc-protected compound **4** was synthesized in solution starting from the known compound **S1**.[12] (Scheme S1). Hydrogenolysis of **S1** was performed in toluene affording free amino compound **2** (99%). The ester function was hydrolyzed in acidic conditions yielding compound **3** (99%). Finally, the Fmoc group was introduced using Fmoc-OSu, in the presence of DIPEA and MeOH/ACN affording **4** (72%).

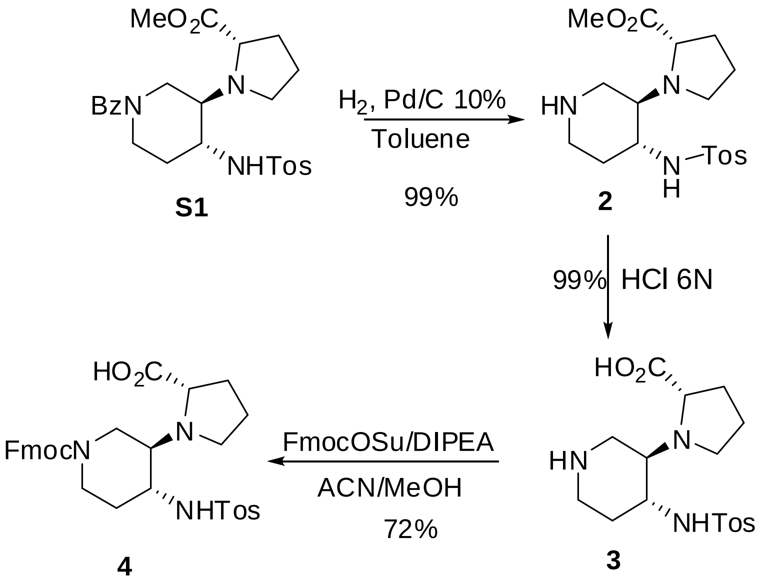

Scheme S1. Synthesis of Fmoc scaffold 4

Methyl 1-[(3R,4R)-4-(4-methylphenylsulfonamido)piperidin-3-yl]-pyrrolidine-2-(S)-carboxylate (2)

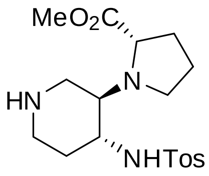

2

Compound **S1** (0.1 g; 0.212 mmol) was dissolved in toluene (20 ml) and Pd/C (10% 0.1 g) was added. The reaction was kept overnight under stirring in hydrogen atmosphere (TLC: CH_2Cl_2/MeOH 20:1). The catalyst was filtered over a «Celite™" pad, and the solvent removed under vacuum, affording compound **2** as a white powder (80 mg, 99%).

$[\alpha]^{25}_D$: -82,14° (*c* 0.28 $CHCl_3$); mp = 122-124°C; IR ν max (KBr): 3436, 3212, 1730cm^{-1}; ^{1}H-NMR (300 MHz, $CDCl_3$) δ ppm: 1.35-1.75 (m, 3H), 1.76-2.25 (m, 4H), 2.42 (s, 3H), 2.30-2.75 (m, 5H), 2.40 (s, 3H), 3.40-3.52 (m, 1H), 3.76 (s, 3H), 6.70 (bs, 1H, exch), 7.27 (d, *J* 2.5, 2H), 7.78 (d, *J* 2.5, 2H); ^{13}C-NMR (75 MHz, $CDCl_3$) δ ppm: 21.7, 24.6, 30.0, 35.3, 44.4, 44.9, 45.1, 52.5, 54.2, 59.9, 62.3, 127.5, 129.6 , 137.7, 143.0, 176.1; $C_{18}H_{27}N_3O_4S$: 381.17 ESI-MS: m/z: 382.2 $[M+H]^+$. HRMS (TOF, ESI, ion polarity positive, MeOH): Calc. Mass for M + $[H]^+$ $C_{18}H_{28}N_3O_4S$ 382.1801, found 382.1801.

1-[(3R,4R)-4-(4-Methylphenylsulfonamido)piperidin-3-yl]-pyrrolidine-2-(S)-carboxylic Acid (3)

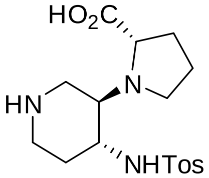

3

Operating in a sealed tube, compound **2** (0.1 g; 0.262 mmol) was dissolved in 6M HCl (10 mL) and heated at 110°C under stirring. After 4 h, the solvent was removed. The crude precipitate was taken up with acetone and a white solid was filtered (95 mg, 99 %).

$[\alpha]^{25}_D$: -2,8°(*c* 1 H_2O); m.p: 170-172 °C; IR ν_{max} (KBr): 3412, 1731 cm^{-1}; ^{1}H NMR (500 MHz, D_2O) δ ppm: 1.56-1.70 (m, 2H), 2.06-2.46 (m, 4H), 2.39 (s, 3H), 2.93-3.40 (m, 4H), 3.71-3.87 (m, 4H), 4.36-4.44 (m,1H), 7.45 (d, *J* 7.8, 2H), 7.80 (d, *J* 8.3, 2H) ^{13}C NMR (125 MHz, D_2O) δ ppm: 21.1, 24.1, 27.4, 28.8, 41.0, 43.1, 50.0, 50.3, 60.3, 67.4, 127.2, 130.9, 135.6, 146.4, 172.0 $C_{17}H_{25}N_3O_4S$: 367.46 ESI-MS: m/z: 368.16$[M+H]^+$ HRMS (TOF, ESI, ion polarity positive, MeOH): Calc. Mass for M + $[H]^+$ $C_{17}H_{26}N_3O_4S$ 368.1644, found 368.1646.

1-[(3R,4R)-1-(((9H-Fluoren-9-yl)methoxy)carbonyl)-4-(4-methylphenylsulfonamido)piperidin-3-yl]-pyrrolidine-2-(S)-carboxylic Acid (4)

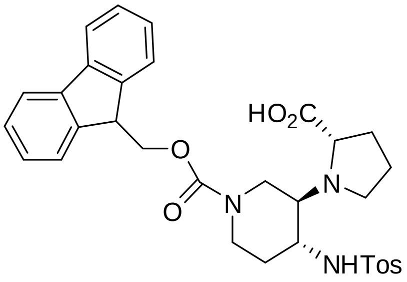

4

Compound **3** (0.12 g, 0.3 mmol) was dissolved in ACN/MeOH (4:1, 10 mL). Fmoc-OSu (0.12 mg, 0.33 mmol) and DIPEA (114 µl, 0.6 mmol) were added to the solution (pH<8). The reaction was left to stir for 3h, (TLC: DCM : AcOEt, 1:1). The solvent was removed under vacuum. The crude residue was resuspended in DCM (30 mL) and washed with H_2O (3 x 30 mL). The organic layer was dried over Na_2SO_4 and the solvent removed. The crude residue was precipitated with AcOEt/Hexane affording compound **4** (0.125 g, 70%) as a white solid.

$[\alpha]^{25}_D$: 29.7°(*c* 1 MeOH); m.p.: 122-127 °C; IR ν_{max}(KBr): 3247, 1740 cm^{-1} ^1H NMR (CD_3OD, 200 MHz) δ 1.56-1.67 (m, 2H), 2.06-2.14 (m, 4H), 2.39 (s,3H), 2.66-2.74 (m, 4H), 3.19-3.29 (m, 2H), 3.67-3.71 (m, 2H), 4.19-4.22 (m, 1H), 4.39-4.80 (m, 3H), 7.20−7.40 (m, 6H), 7.54-7.57 (m, 2H), 7.65-7.80 (m, 4H); ^{13}C NMR ($CDCl_3$, 50 MHz) δ 20.3, 24.4, 25.1, 29.7, 31.1, 41.2, 41.6, 52.1, 54.6, 60.8, 66.3, 67.1, 119.8, 124.5, 127.1, 127.2, 127.7, 129.7, 137.5, 141.5, 144.0, 155.2, 174.7 $C_{32}H_{35}N_3O_6S$: 589.7 ESI-MS: m/z: 590.8 $[M+H]^+$ HRMS (TOF, ESI, ion polarity positive, MeOH): Calc. Mass for M + $[H]^+$ $C_{32}H_{36}N_3O_6S$ 590.2325, found 590.2321; Calc. Mass for M + $[Na]^+$ $C_{32}H_{35}N_3O_6NaS$ 612.2144, found 612.2140.

Synthesis of Peptides SRE1-3, G1-3

Peptides **SRE1-3** were synthesized on Rink-amide resin (0.72 loading) using standard conditions[13] (AA/HOBT/HBTU/DIPEA, 5:5:5:10); 1 h coupling and then 20% piperidine in DMF for Fmoc deprotection).

Regarding peptides **G1-3**, the coupling of **4** (1.5 eq) was performed on the peptide growing chain linked to rink-amide resin using HOBT and HBTU (1.5 eq) and DIPEA (3 eq), and standing the mixture under shaking overnight. Compounds **G1a, G2a, G3** (Scheme S2), **SRE1-3** were finally acetylated on resin using Ac_2O (10 eq) and DIPEA (10 eq). The cleavage was then performed using reagent K[14] (trifluoroacetic acid/phenol/water/thioanisole/1,2-ethanedithiol; 82.5:5:5:5:2.5) for 180 min. After the cleavage, the peptides were precipitated and washed using ice-cold anhydrous ethyl ether. The

peptides was purified by RP-HPLC using a gradient elution of 95–30% solvent A (solvent A: water/acetonitrile/trifluoroacetic acid 95 : 5 : 0.1; solvent B: water/acetonitrile/trifluoroacetic acid 5 : 95 : 0.1) over 20 min at a flow rate of 20 mL/min^{-1}. The purified peptides were freeze-dried and stored at 0 °C.

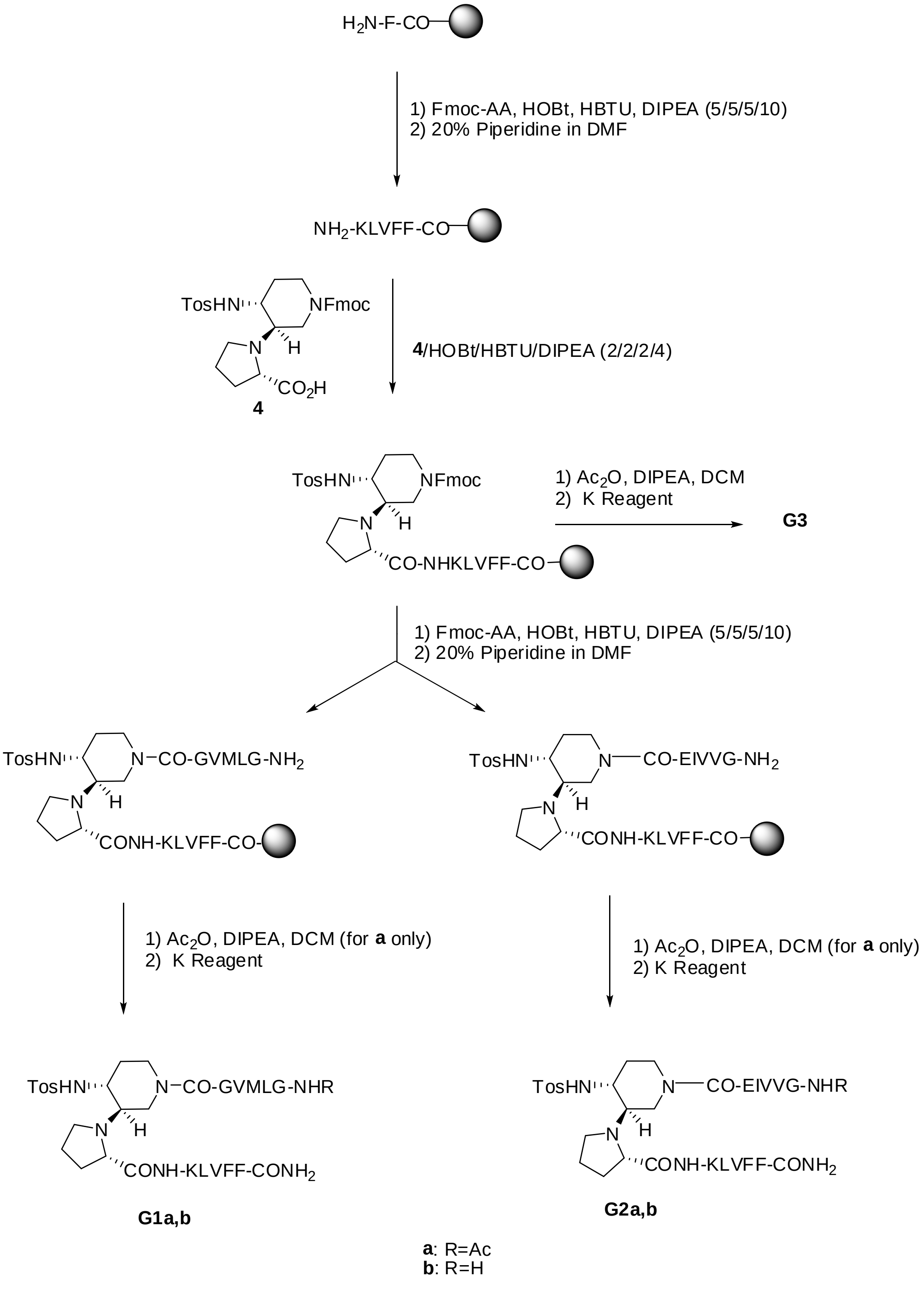

Scheme S2. Synthesis of **G1**, **G2**, **G3**

Table 5. HPLC retention time and MW of peptides **SRE1-3**, **G1-3**.

Compound	HPLC rt[a]	Calculated MW	Found MW
**SRE1**	16.192	516.66	539.8 (MW + Na^+)
**SRE2**	15.167	556.66	579.8 (MW + Na^+)
**SRE3**	19.700	693.89	694.6 (MW + 1)<br>716.6 (MW + Na^+)
**G1a**	20.725	1500.47	1501.8 (MW +1)<br>751.9 (MW + 2)/2
**G2a**	22.292	1539.65	1540.8 (MW + 1)<br>770.5 (MW + 2)/2
**G1b**	20.492	1458.91	1481.2 (MW + Na^+)
**G2b**	21.033	1497.23	1498.9 (MW + 1)<br>750.7 (MW + 2)/2
**G3**	21.050	1042.4	1043.6 (MW + 1)<br>1065.8 (MW + Na^+)

[a] analytical HPLC gradient elution: of 95–30% solvent A (solvent A: water/acetonitrile/trifluoroacetic acid 95 : 5 : 0.1; solvent B: water/acetonitrile/trifluoroacetic acid 5 : 95 : 0.1) over 40 min at a flow rate of 0.8 mL/min^{-1}.

Circular Dichroism of compounds G1a and G2a

Solutions of **G1a** and **G2a** were prepared in MeOH (50 µM, 1.5 mL). CD spectra were obtained from 195 to 250 nm with a 0.1 nm step and 1 s collection time per step, taking three averages. The spectrum of the solvent was subtracted to eliminate interference from cell, solvent, and optical equipment. The CD spectra were plotted as mean residue ellipticity θ (degree x cm^2 x $dmol^{-1}$) versus wave length λ (nm). Noise-reduction was obtained using a Fourier-transform filter program.

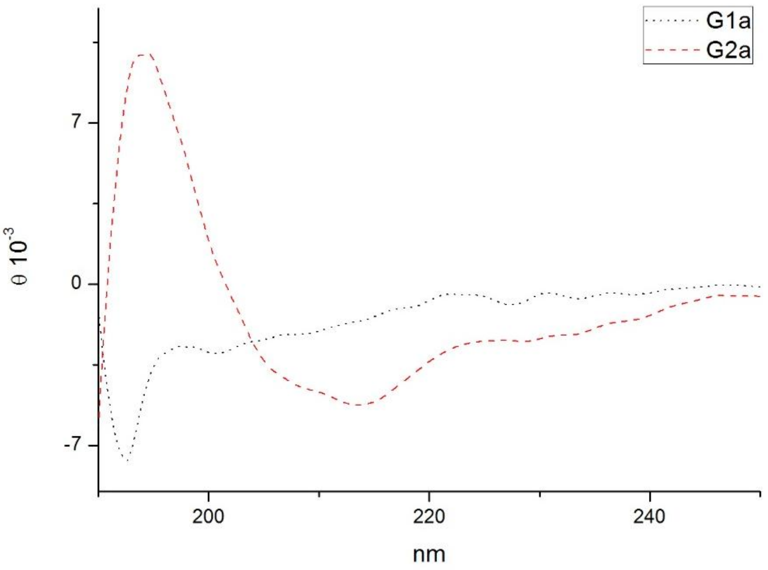

Figure S2. CD spectra of **G1a** and **G2a** (50 µM in MeOH)

NMR discussion for compounds G1a and G2a

All experiments (^{1}H, COSY, TOCSY, NOESY and ROESY) were recorded in MeOH (4 mM) at 500 MHz. As the piperidine scaffold is present as a mixture of conformers, many signals are overlapped in the aliphatic region. Only chemical shifts of the piperidine main conformer are reported.

Compound **G1a** is present in solution as a dynamic equilibrium between two different β-hairpin structures (2:1 ratio), characterized by a different alignment of the two peptide arms (compounds **G1a-1** is the main isomer, and **G1a-2** is the minor one). On the other hand, compound **G2a** is present in solution as a stable single β-hairpin conformation characterized by a peptide arms alignment similar to **G1a-2**.

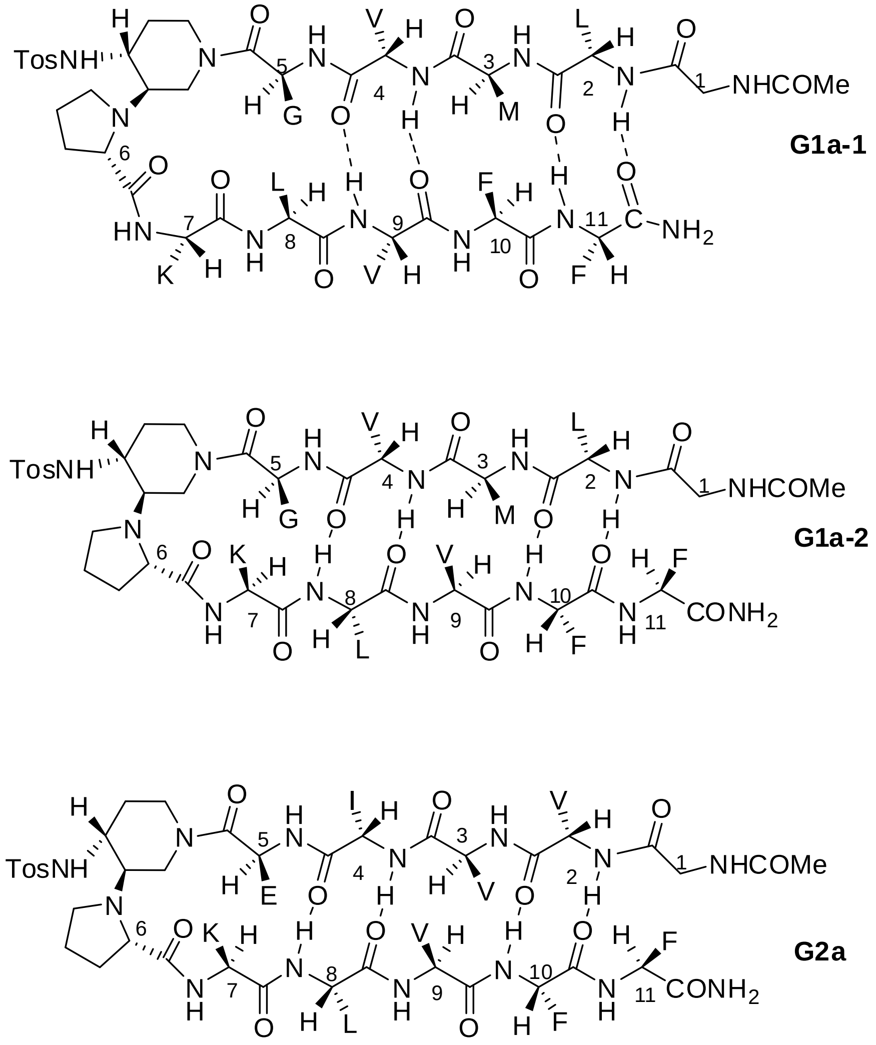

Figure S3. Structures of compounds **G1a-1**, **G1a-2**, and **G2a**

NMR data and discussion for peptide G1a

NMR characterization of **G1a** has been very complex but the chemical shifts of two main isomers (2:1 ratio), named **G1a-1** (Table S6) and **G1a-2** (Table S7), were assigned.

The presence of two conformers of **G1a** is proved by several negative NH/NH ROEs (Figure FS4 in comparison with Figure FS13 related to **G2a**). This observation indicates a dynamic equilibrium between different conformers.[15] Interestingly, a strong intrastrand ROE was found between NH_{Val4} of **G1a-2** and CH_{Val4} of **G1a-1** one (Figure FS6B), suggesting that Val-4 is probably involved in the dynamic switch between the two isomers.

ROESY experiments confirmed the presence of a turn structure in both **G1a-1** and **G1a-2** isomers. Spatial proximity was indeed observed between the piperidine moiety of scaffold **S1** (H-2 and H-6) with both proline and Gly-5 (Figure FS5), confirming the reported data for model sequences.[12]

A complete set of CH/NH(i, i+1) ROEs is present for isomer **G1a-1** (except for Val-4/Met-3 and Leu-2/Gly-1; Figures FS6A and FS6C). CH/NH interstrand ROEs (Figures FS5A and FS6) between NH_{Val4}/CH_{Leu8}, NH_{Phe11} with both CH_{Gly1} and MeCO and NH_{Phe11}/CH_{Met3} are present. The last ROE is of particular relevance to demonstrate that the β-hairpin of **G1a-1** is characterized by a different hydrogen bond network with respect to **G1a-2** minor isomer.

The presence of a β-hairpin structure is further confirmed by positive ΔδαH shift values.[16] (Table TS8 and Figure FS8). These values are smaller with respect to **G1a-2** and **G2a** indicating that the hairpin conformation of **G1a-1** is not very stable. Only Met-3 is characterized by a negative ΔδαH value. This is probably due to the anisotropic effect of the aromatic ring of Phe-11,[17] that faces Met-3, as demontrated by Roesy experiment.

The minor conformer **G1a-2** showed higher ΔδαH values with respect to those of **G1a-1** (Table TS8). Of relevance, the positive value of methionine, indicating its different sterical environment. A complete set of CH/NH(i, i+1) ROEs are present, except for NH_{Val9} and CH_{Leu8} (Figures FS6B and FS6C). Interstrand ROEs were found between NH_{Val4}/CH_{Leu8}, NH_{Phe11}/MeCO and NH_{Leu2}/CH_{Val9}, indicating the formation of a β-hairpin characterized by the same H-bond network proposed for **G2a** (see below, Figures FS6B and FS7).

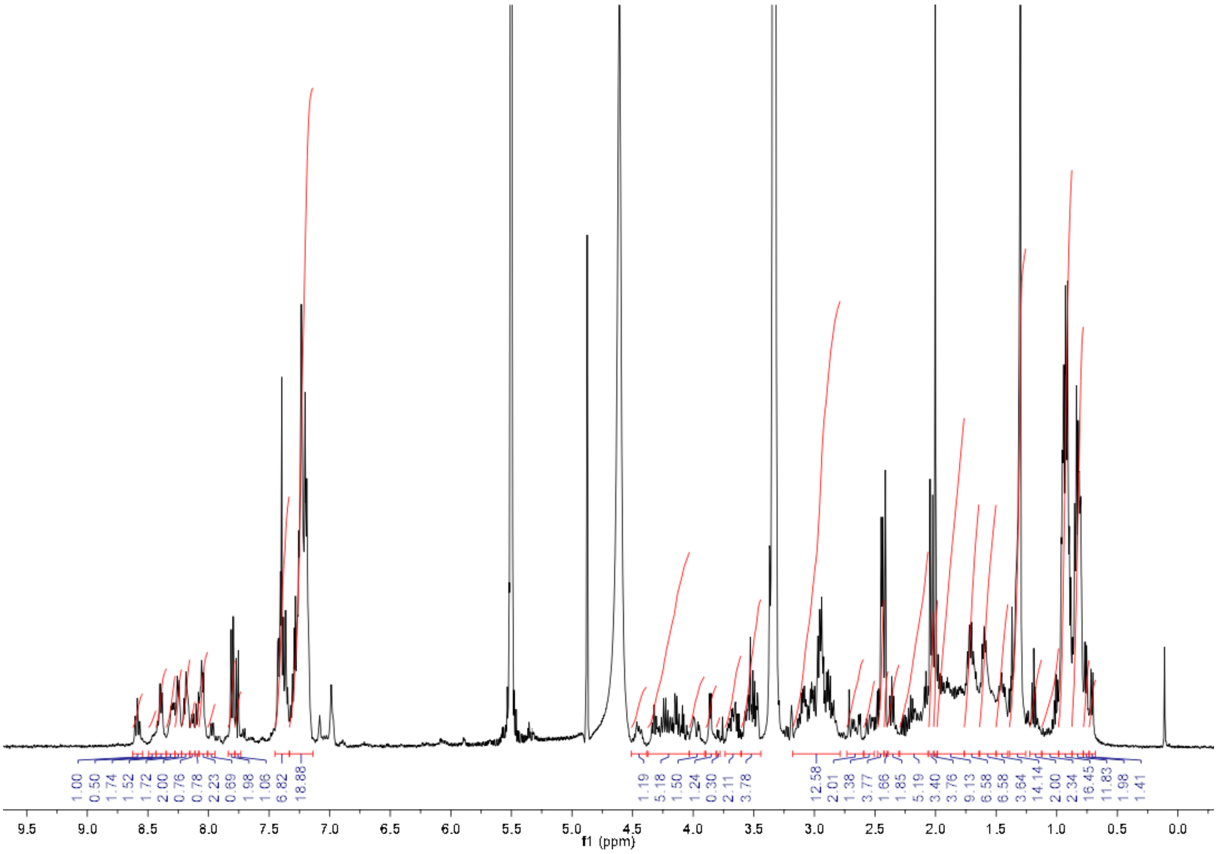

Table S6. ^{1}H NMR chemical shifts of G1a-1

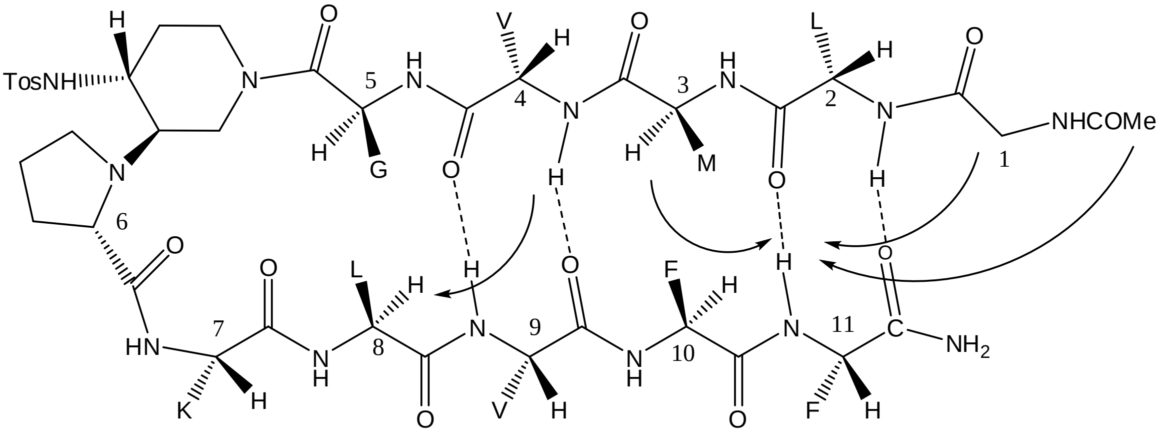

G1a-1

Residue	NH	α-H	β-H	Other	Roesy[a]
MeCO				2.00	NH_{Phe11}(w)[b]
Gly-1	8.17	3.97/3.78			NH_{Phe11}
Leu-2	8.38	4.26	1.84	0.80, 0.83	
Met-3	8.24	3.86	1.97, 2.04	2.45, 2.54 Me: 2.05[b]	NH: CH_{leu2} (m) CH: NH_{Phe11} (m)[c]
Val-4	8.08	4.13	1.96	0.83, 0.81	NH: CH_{Leu8} (m)[c] CH: °NH_{Val4}[d]
Gly-5	8.04	4.10/3.99			NH: CH_{Val4}(s), H-6_{Sc}(s)
Scaffold			H-2: 4.31, H-2': 3.69		H-2: H-2'(s), H-4(m),

			H-3: 3.49; H-4: 2.47<br>H-5: 2.36, 1.63<br>H-6: 4.43, H-6’: 2.85<br>NH: 6.07<br>Arom: 7.80*o*, 7.46*m*;<br>Me: 2.44		H-5$_{Pro6}$(m);<br>H-4: H-2 (m), H-5$_{Pro6}$(s), H-5’$_{Pro6}$(m)<br>H-5’: H-5(s)<br>H-6: CH$_{Gly5}$(s),<br>NH: H-4(w), H-6’ (w)
Pro-6		3.68	H-5: 2.96, H-5’: 2.20; H-4: 1.77, 1.57; H-3: 2.17, 1.89		H-5: H-3(2.17), H-2$_{Sc}$(4.31), H-4$_{Sc}$(s)<br>H-5’: H-4$_{Sc}$(m)
Lys-7	8.59<br>(*J* 9.2)	4.47	1.94	Hγ: 1.53<br>Hδ: 1.44, 1.71<br>Hε: 2.95<br>NH_3^+ 3.68[b]	Hε: Hδ 1.71(s)
Leu-8	8.19	4.23	1.81	0.84	NH: CH$_{Lys7}$ (s)<br>CH: NH$_{Val4}$ (m) [c]
Valine-9	8.07	4.19	1.96	0.76/0.81	NH: CH$_{Leu8}$(s)
Phe-10	8.13<br>(*J* 7.9)	4.68	3.03, 2.87	Ar:7.20	NH: CH$_{Val9}$(m)
Phe-11	8.17	4.66	3.08, 2.93	Ar:7.24	NH: CH$_{Phe10}$(vw)<br>NH:[c] CH$_{Met3}$(m), MeCO (w), CH$_{Gly1}$(w),
NH_2	6.98				

[a]Only significant ROEs are reported; [b]Tentatively assigned; [c]Intrastrand Roesy. [d]A Roesy between NH of Val-4 of isomer °**G1a-2** and CH of Val-4 of isomer ***G1a-1** was tentatively assigned.

Table S7. **^{1}H NMR chemical shifts of G1a-2**

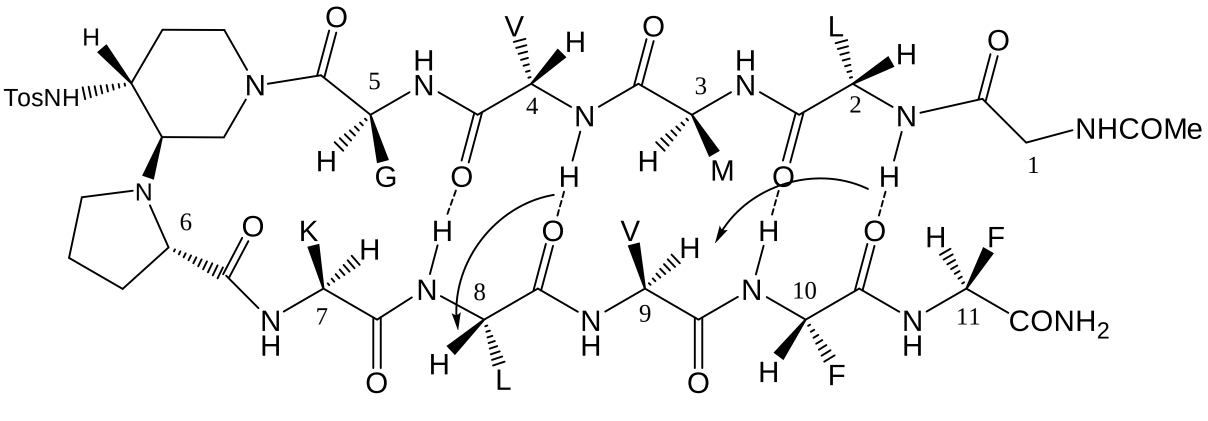

G1a-2

Residue	NH	α-H	β-H	Other	ROESY[a]
MeCO				2.02[b]	NH$_{Phe10}$(w)[b]
Gly-1	8.18	3.97/3.78			
Leu-2	8.29	4.46	1.71,1.82	0.71, 0.82	CH: NH$_{Gly1}$(s)<br>NH: CH$_{Val9}$(m)[c]
Met-3	8.39	4.57	1.98,1.92	2.38, 2.45<br>Me: 2.02[b]	NH: CH$_{Leu-2}$(w)

Val-4	8.05	4.32	2.12	0.92	NH: CH$_{Met}$(vs), CH$_{Leu8}$ (m)[c], *CH$_{Val4}$[d]
Gly-5	8.03	4.10/3.95			NH: CH$_{Val4}$(s)
Scaffold[d]			H-2: 4.31, H-2': 3.54<br>H-3: 3.51; H-4: 2.24<br>H-5: 2.36, 1.69<br>H-6: 4.44, 2.86<br>NH 6.06<br>Arom: 7.76*o*, 7.46*m*;<br>Me: 2.42		
Pro-6[d]		3.68	H-5: 2.96, H-5': 2.21, H-4: 1.73, 1.59, H-3: 1.71, 1.87		
Lys-7	8.57<br>(*J* 9.0)	4.44	1.93	Hγ: 1.53<br>Hδ: 1.44, 1.72<br>Hε: 2.96	
Leu-8	7.97<br>(*J* 8.3)	4.24	1.80	0.81	NH: CH$_{Lys7}$ (vw)<br>CH: NH$_{Val4}$ (m)[c,a]
Valine-9	8.40	4.33	2.08	0.93	CH: NH$_{Leu2}$ (m)[c]
Phe-10	8.25	4.58	3.09, 2.92	Ar: 7.23	NH: CH$_{Val9}$(m), MeCO (w),[c] Ar (7.23)
Phe-11	8.31	4.62	3.03, 2.87	Ar: 7.20	NH: CH$_{Phe10}$(w)<br>Ar: 7.20
NH$_2$	7.08				

[a]Only significant Roesy are reported; [b]Tentatively assigned; [c]Interstrand Roesy. [d]A Roesy between NH of Val-4 of isomer °**G1a-2** and CH of Val-4 of isomer ***G1a-1** was tentatively assigned.

Table S8. δ CH NMR data for **G1a** peptide

AA	CHα[a] random	δ CHα **G1a-1**	Δδ CHα **G1a-1**	δ CHα **G1a-2**	Δδ CHα **G1a-2**
Gly-1	3.97	3.97/3.78	0/-0.19	3.97/3.78	0/-0.19
Leu-2	4.17	4.26	0.09	4.46	0.29
Met-3	4.52	3.86	-0.64	4.57	0.05
Val-4	3.95	4.13	0.18	4.32	0.37
Gly-5	3.97	4.10/3.99	0.13/0.02	4.10/3.95	0.13/0.02
Pro-6	4.44	3.68	-0.76	3.68	-0.76
Lys-7	4.36	4.47	0.11	4.44	0.08
Leu-8	4.17	4.23	0.06	4.24	0.07
Val-9	3.95	4.19	0.24	4.33	0.38
Phe-10	4.66	4.68	0.02	4.58	-0.08
Phe-11	4.66	4.66	0.00	4.62	-0.04

[a]Difference between Hα chemical shift values in the random coil and the values determined experimentally[16]

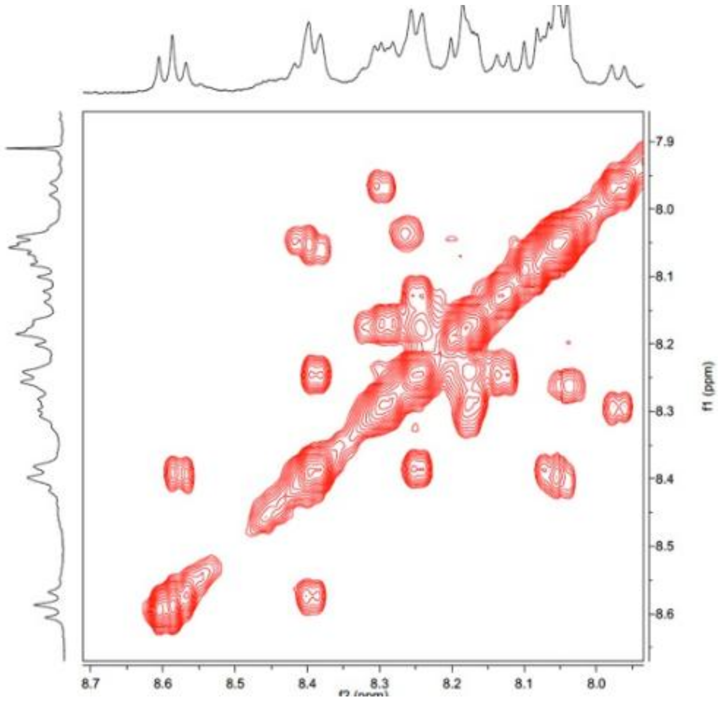

Figure S4. NH/NH region in the Roesy experiment of **G1a** (500 MHz, 200 ms)

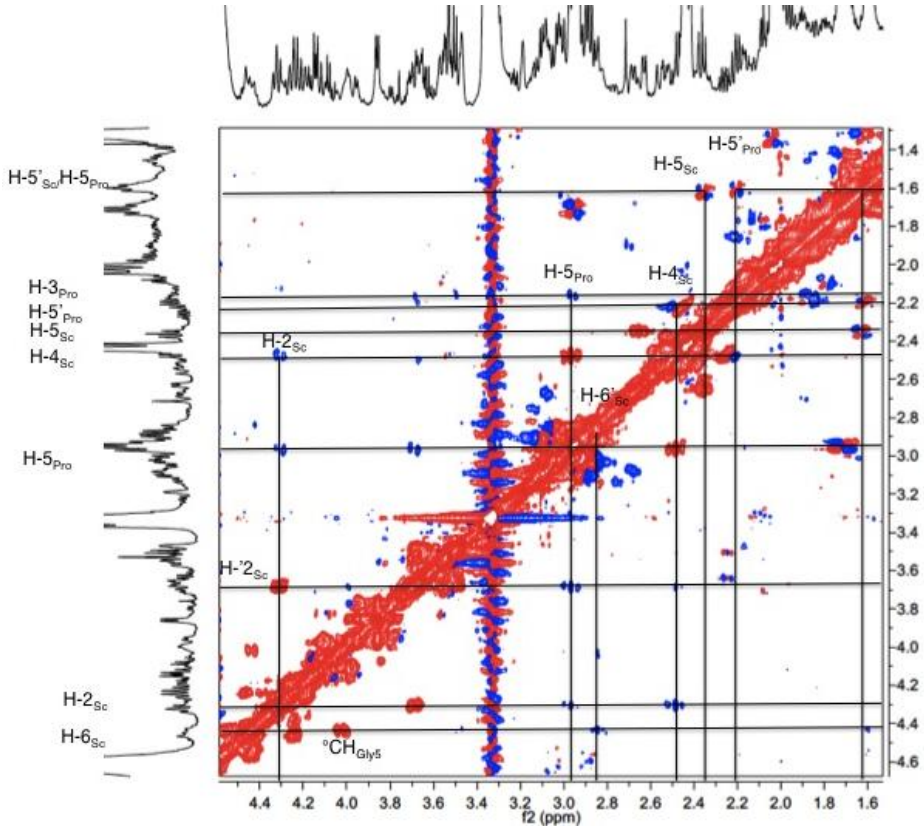

Figure S5. CH region of **G1a** in the Roesy experiment (500 MHz, 200 ms). The figure watoo small I have increased its size. A scheme of the numerotation of the scaffold protons would help

A)

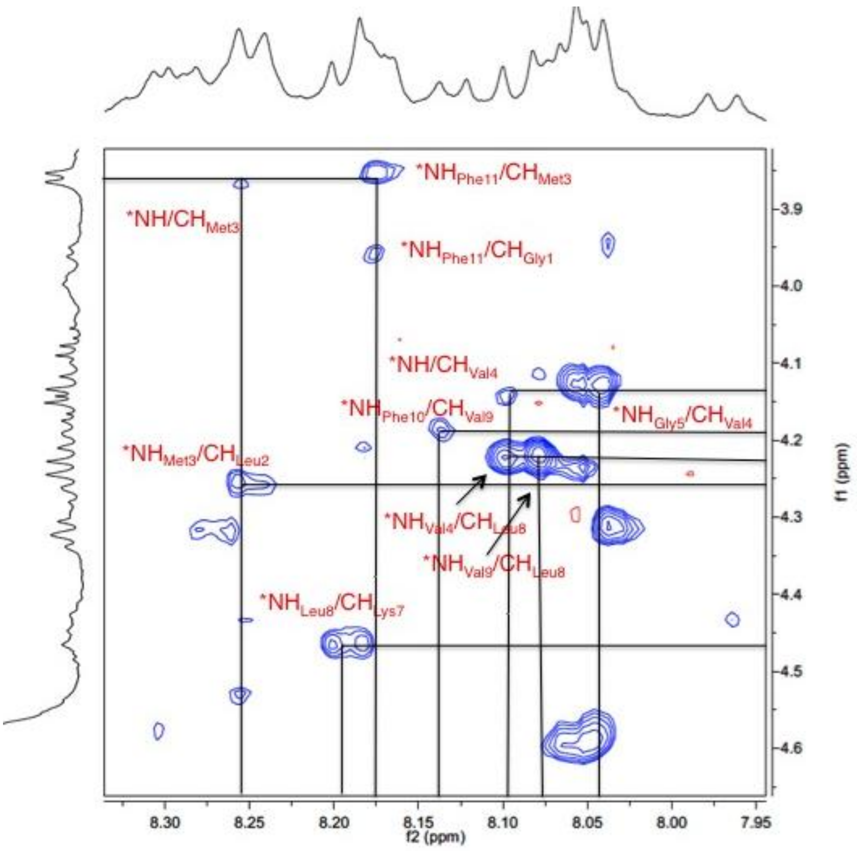
*NH$_{Phe11}$/CH$_{Met3}$
*NH/CH$_{Met3}$
*NH$_{Phe11}$/CH$_{Gly1}$
*NH/CH$_{Val4}$
*NH$_{Phe10}$/CH$_{Val9}$
*NH$_{Gly5}$/CH$_{Val4}$
*NH$_{Met3}$/CH$_{Leu2}$
*NH$_{Val4}$/CH$_{Leu8}$
*NH$_{Val9}$/CH$_{Leu8}$
*NH$_{Leu8}$/CH$_{Lys7}$
f1 (ppm)
f2 (ppm)

B)

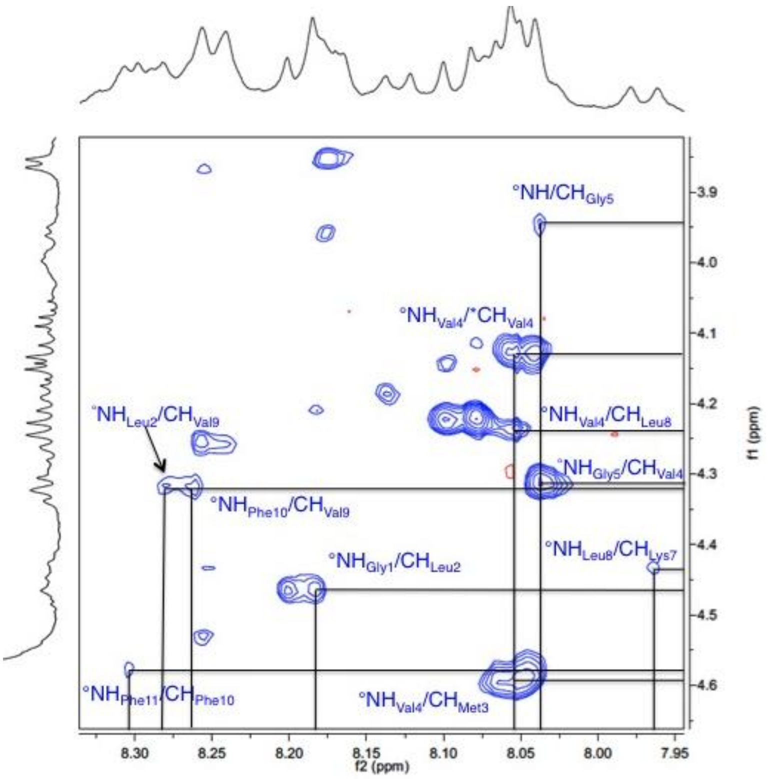
°NH/CH$_{Gly5}$
°NH$_{Val4}$/*CH$_{Val4}$
°NH$_{Leu2}$/CH$_{Val9}$
°NH$_{Val4}$/CH$_{Leu8}$
°NH$_{Gly5}$/CH$_{Val4}$
°NH$_{Phe10}$/CH$_{Val9}$
°NH$_{Gly1}$/CH$_{Leu2}$
°NH$_{Leu8}$/CH$_{Lys7}$
°NH$_{Phe11}$/CH$_{Phe10}$
°NH$_{Val4}$/CH$_{Met3}$
f1 (ppm)
f2 (ppm)

C)

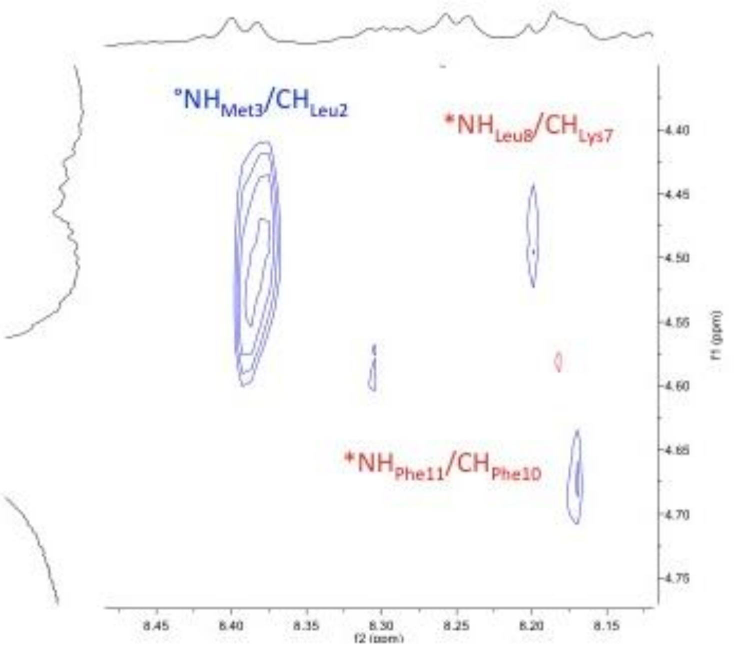

Figure S6. δ NH/CH region in the Roesy experiment (500 MHz, 200 ms): A): **G1a-1;** B): **G1a-2**; C) Zoom of NH/CH region, red: **G1a-1**, blue:**G1a-2**.

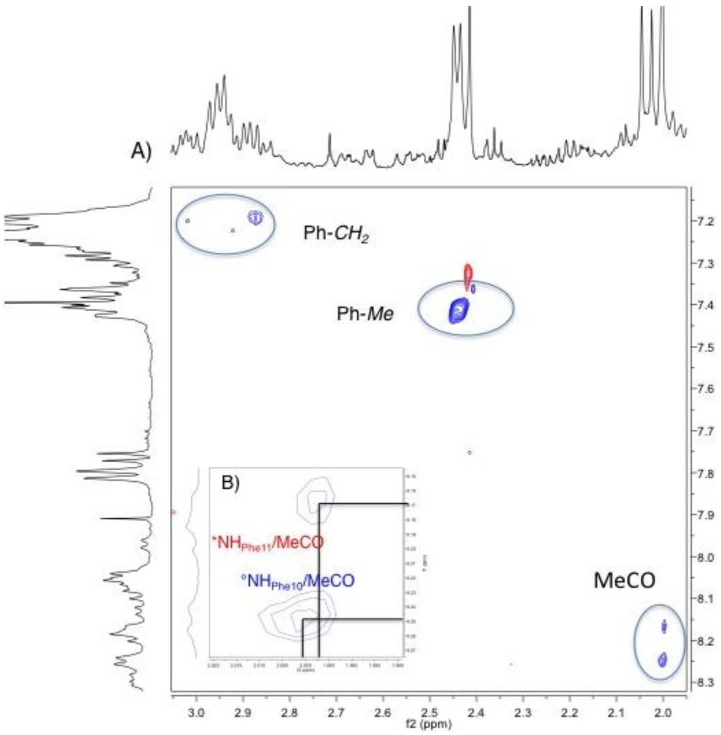

Figure S7. A) Arom/NH and Me region of **G1a** in the Roesy experiment (500 MHz, 200 ms). B) Zoom of the Arom/MeCO region. Red: **G1a-1**; Blu: **G1a-2**.

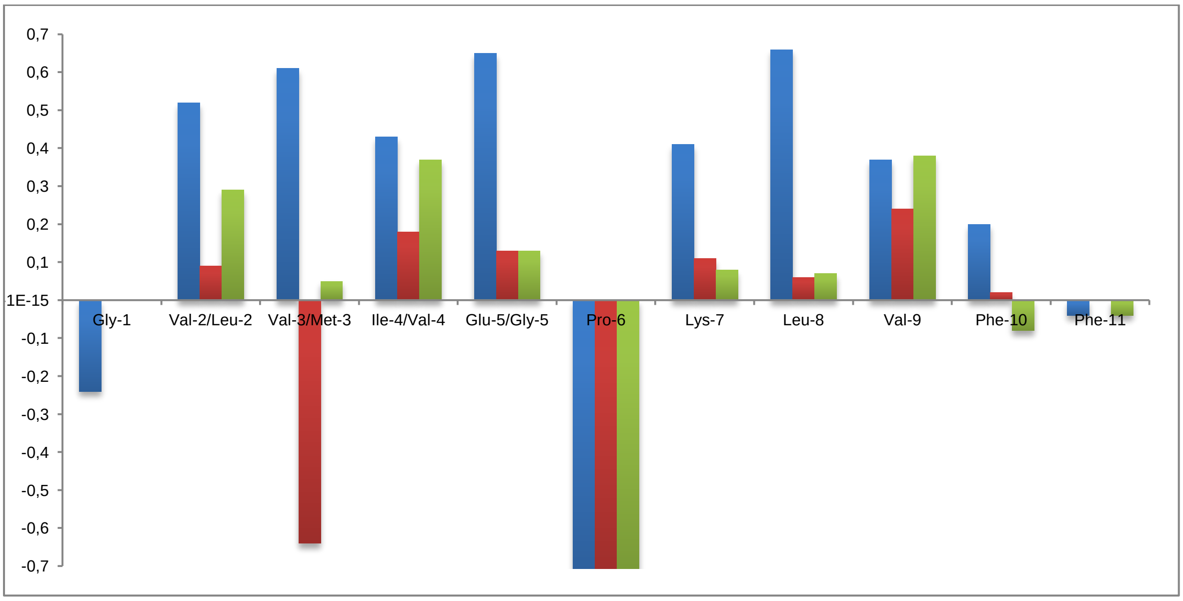

Figure S8. NMR analysis. Plot of difference between Hα chemical shift values in the random coil[16] and the values determined experimentally for **G2a** (blue) and isomers **G1a-1** (red) and **G1a-2** (green) in CD_3OH at 298 K. 5:E.[18]

NMR data and discussion for peptide G2a

NOESY and ROESY experiments on compound **G2a** evidenced strong CH/NH(i, i+1)ROEs (Figure FS10) and the formation of a turn in the region containing **S1** scaffold. Spatial proximity between the piperidine moiety of scaffold **S1** (H-2 and H-6) with both proline and Glu-5 (Figure FS11) was observed.

A cross-strand Noe was detected between CH_{Gly-} Gly1?? and the phenyl ring of Phe-10 (Figure FS12). However, no other not ambiguous NOEs were observed between cross-strand residues due to peaks overlapping among them or overlapped with the solvent signals.

The profiles of ΔδαH conformational shift values ($\Delta\delta_{observed}$ - $\Delta\delta_{random\ coil}$, ppm; Table TS10 and Figure FS8), as well as a deviation of more than 0.1 ppm from random coil for several successive residues exhibited by peptide **G2a**, are consistent with those of the target β-stranded antiparallel β-sheet.[18] Furthermore the separation of the Gly resonances indicates a β-hairpin structure as reported in the literature.[19]

A further confirmation is given by $^3J_{HN/CH}$ coupling constant distributions that are commonly used for identifying secondary structure in the NMR structure determination.[20] Values higher

than 8 Hz characterize a β strand, as observed for **G2a** peptide. As shown in Figure FS9 and Table TS10, positive difference between $^{3}J_{HN/CH\alpha}$ values in the random coil and the values determined experimentally were found. As an exception, Glu-5 is characterized by a lower J value that is justified by a sharp change in backbone direction indicative of a β-turn. [21]

Table S9. ^{1}H NMR data for **G2a**

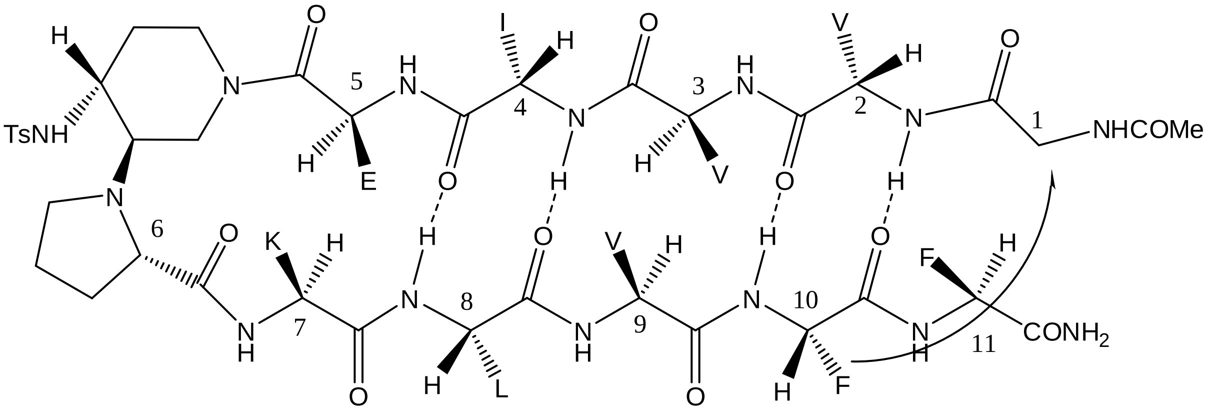

G2a

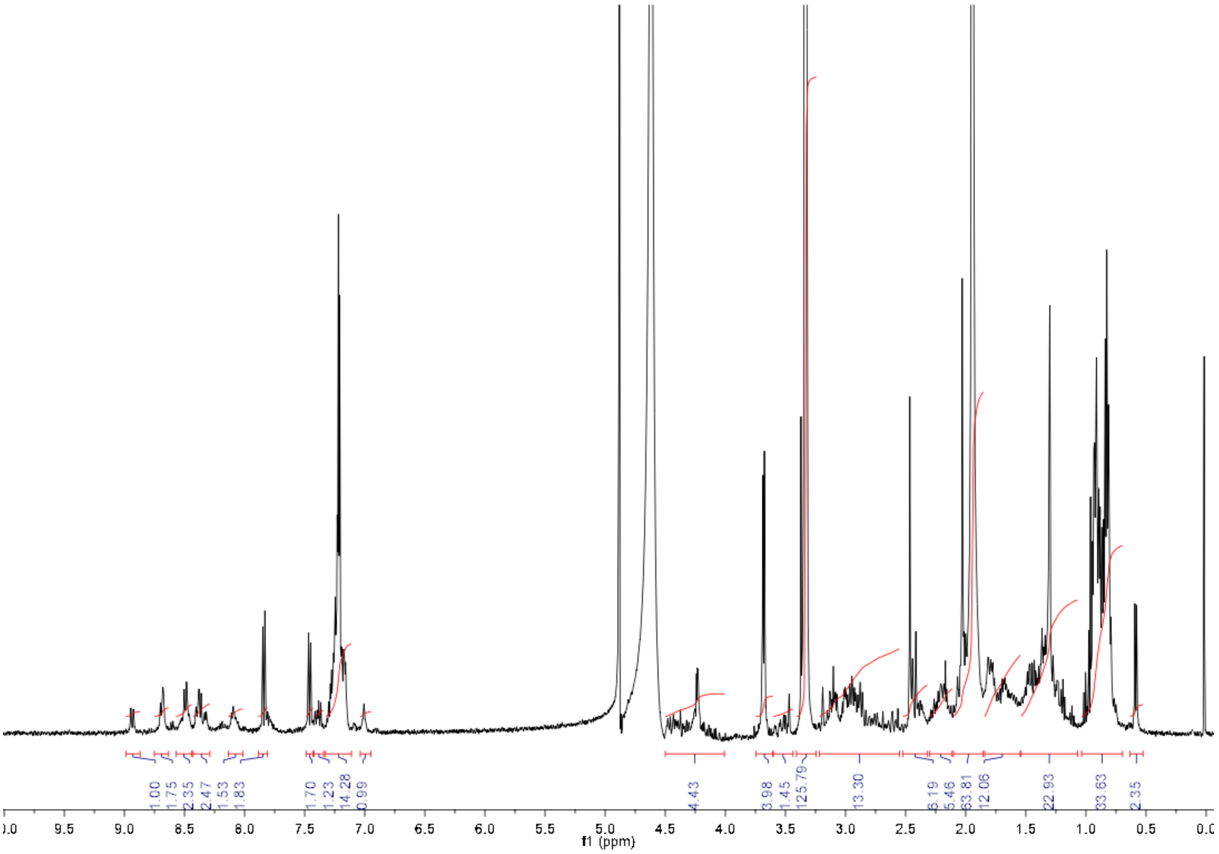

Residue	NH	α-H	β-H	Other	Roesy[a]
MeCO				2.02	
Gly-1	8.09 (J 7.8[c])	4.19/3.57 (J 19)			CH_2: NH_{Val2}(vw), Ar_{Phe10}(7.21)[b]
Val -2	8.49 (J 11.7[c])	4.47	2.00	0.92	NH: H_{Gly1}(vw)
Val-3	8.39 (J 9.5 J 9.6[c])	4.56	1.93	0.89, 0.81	NH: H_{Val2}(s) CH: NH_{Ile4}(s)
Ile-4	8.94 (J 10.3 J 10.6[c])	4.38	1.83	0.88	NH: H_{Val3}(s)
Glu-5	8.67 (J 6.1 J 6.3[c])	4.94	2.19, 1.95		NH: H_{Ile4}(s) CH: H_2/H_{6Scaf} (4.43-4.46 region, s)
Scaffold[d]			H-2: 4.45, H-2’: 3.13 H-3: 2.89; H-4: 2.80 H-5: 2.40, H-5’: 1.24 H-6: 4.42, H-6’: 2.60 Arom: 7.85*o*, 7.46*m*; Me: 2.46		H-2: H-2_{Pro6}(w), H_{Glu5}(w) H-2’: H-2(s), H-3(s) H-5: H-5’(s), H-4(w) H-6: H-6’(vs), H_{Glu5}(m) Arom (7.46): Me (2.46)
Pro-6		3.68	H-3: 1.92 H-4: 1.80, 1.66 H-5: 2.39		H-2: NH_{Lys7}(s), H-4(w), H-3(w), H-2_{Scaf}(w)
Lys-7	8.36 (J 9.1 J 9.6[c])	4.77	1.78	Hγ/Hδ: 1.62, 1.48 Hε: 3.0	NH: H-2_{Pro6}(s) CH: NH_{Leu8}(s)
Leu-8	8.48 (J 9.0[c])	4.73	1.59, 1.50	0.58, 0.83, 0.93	NH: CH_{Ly7}(s)
Val-9	8.69 (J 10.1 J 11.0[c])	4.32	1.96	0.83	NH: CH_{Leu8}(s)
Phe-10	8.46 [e]	4.86	3.09, 2.86	Ph: 7.26-7.15	NH: CH_{Val9}(m) Ar(7.21): CH(m), CH_2(2.86,s), CH_{Gly1}(vw) [b]
Phe-11	8.31 (J 8.2)	4.62	3.11, 2.95	Ph: 7.26-7.15	Ar (7.22): CH(σ), CH_{2Phe11} (3.11,s)
NH_2	6.91 7.09				

[a]Only significant Roesy are reported; [b]Intrastrand Roesy; [c]Calculated *via* COSY experiment; [d]Main scaffold. [e]Not detectable

Table S10. NH/CH NMR data for **G2a** peptide

AA	$^3J_{HN/H\alpha}$ [b] random	$^3J_{HN/H\alpha N}$	ΔJ	δ CHα [a] random	δ CHα	$\Delta\delta$ CHα
Gly-1	5.9	7.8 [c]	1.9	3.97	4.19/3.57	0.24/-0.40
Val-2	7.7	11.7 [c]	4.0	3.95	4.47	0.52
Val-3	7.7	9.5	1.8	3.95	4.56	0.61
Ile-4	7.6	10.3	2.7	3.95	4.38	0.43
Glu-5	6.8	6.1	-0.7	4.29	4.94	0.65
Pro-6	-		-	4.44	3.68	-0.76
Lys-7	7.1	9.1	2.0	4.36	4.77	0.41
Leu-8	7.1	9.0 [c]	1.9	4.17	4.73	0.66
Val-9	7.7	10.1	2.4	3.95	4.32	0.37
Phe-10	7.5	[d]		4.66	4.86	0.20
Phe-11	7.5	8.2	0.7	4.66	4.62	-0.04

[a] Difference between Hα chemical shift values in the random coil and the values determined experimentally[16]; [b] *J* values derived from the COIL data set as listed in Smith et al.[20b] [c] Calculated *via* COSY experiment. [d] Not determined.

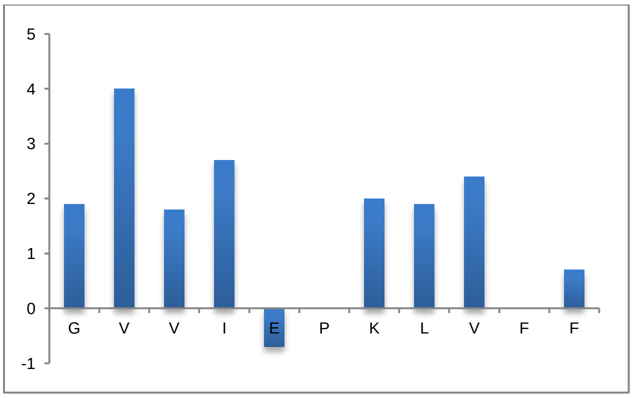

Figure S9. NMR analysis. Plot of difference between $^3J_{HN/CH\alpha}$ values in the random coil and the values determined experimentally for **G2a**.

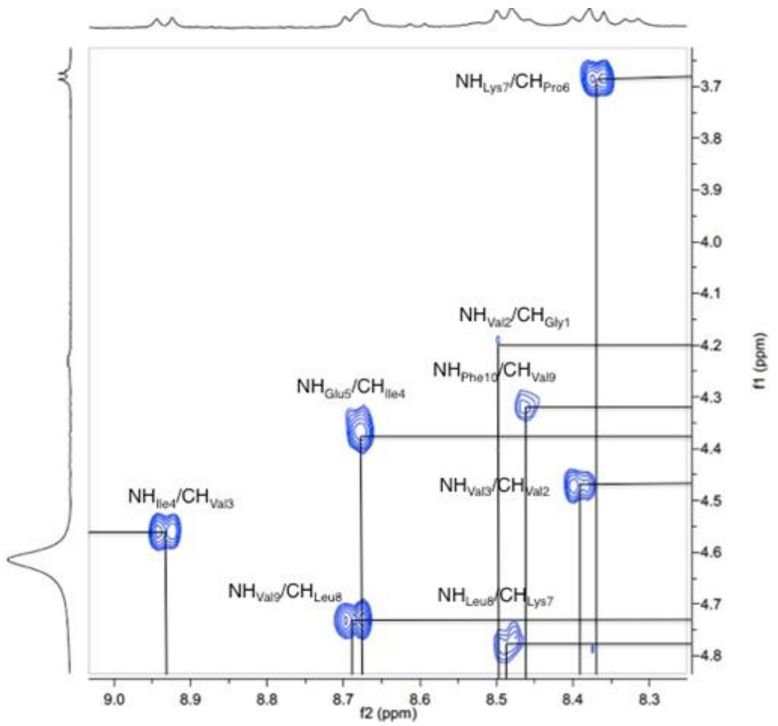

Figure S10. NH/CH region of **G2a** in the Roesy experiment (500 MHz, 200 ms).

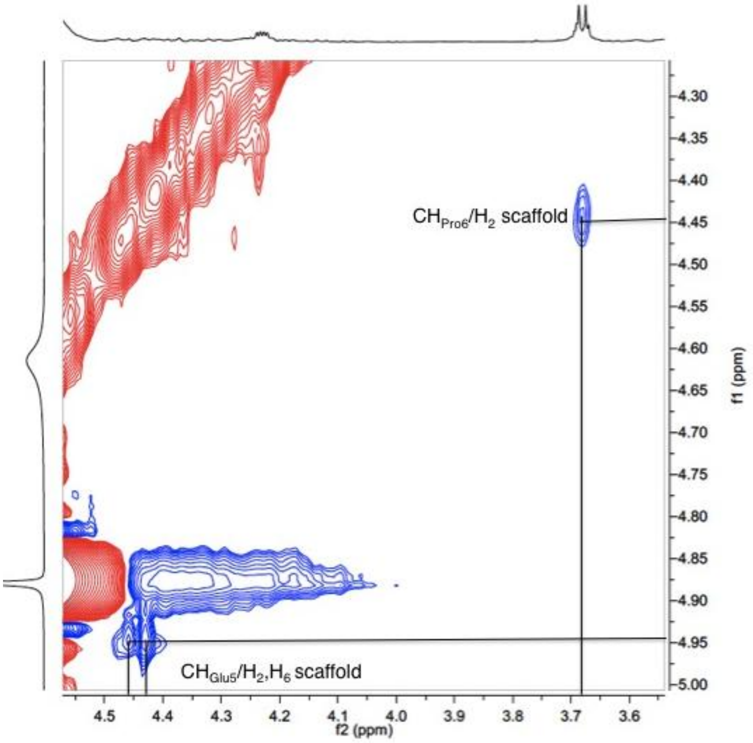

Figure S11. CH region of **G2a** in the Roesy experiment (500 MHz, 200 ms)

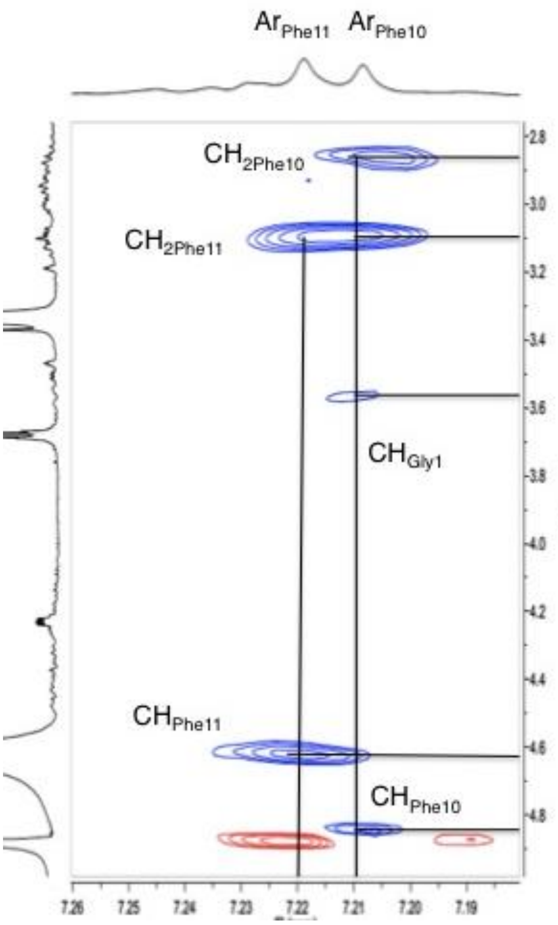

Figure S12. Zoom of CH/Aromatic region of **G2a** in the Roesy experiment (500 MHz, 200 ms).

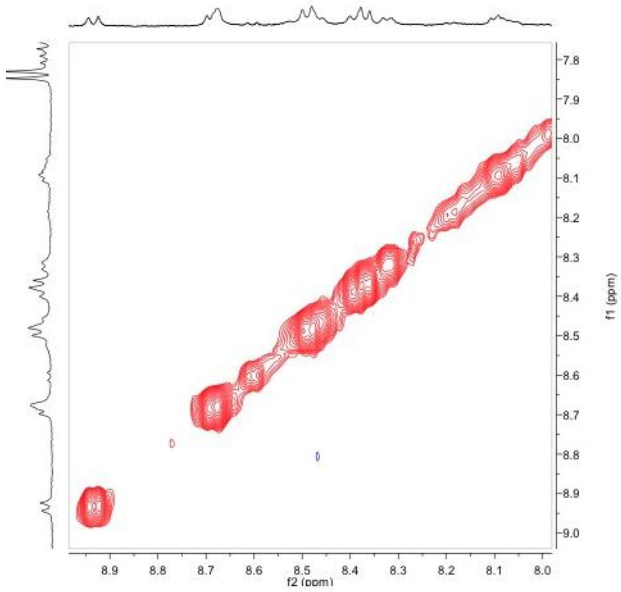

Figure S13. NH region of **G2a** in the Roesy experiment.

Thioflavin-T assay (A$\beta_{1\text{-}42}$)

Thioflavin T was obtained from Sigma. A$\beta_{1\text{-}42}$ was purchased from American Peptide. The peptide was dissolved in an aqueous 1% ammonia solution to a concentration of 1 mM and then, just prior to use, was diluted to 0.2 mM with 10 mM Tris-HCl, 100 mM NaCl buffer (pH 7.4). Stock solutions of β-hairpin mimics were dissolved in DMSO with the final concentration kept constant at 0.5% (v/v).

Thioflavin-T fluorescence was measure to evaluate the development of A$\beta_{1\text{-}42}$ fibrils over time using a fluorescence plate reader (Fluostar Optima, BMG labtech) with standard 96-wells black microtiter plates. Experiments were started by adding the peptide (final A$\beta_{1\text{-}42}$ concentration equal to 10 µM) into a mixture containing 40 µM Thioflavin T in 10 mM Tris-HCl, 100 mM NaCl buffer (pH 7.4) with and without the tested compounds at two different concentrations (100 and 10 µM) at room temperature. The Th-T fluorescence intensity of each sample (performed in duplicate or triplicate) was recorded with 440/485 nm excitation/emission filters set for 42 hours performing a double orbital shaking of 10 s. before the first cycle. The fluorescence assays were performed between 2 and 4 times on different days, with two different batches of peptide. The ability of compounds to inhibit A$\beta_{1\text{-}42}$ aggregation was assessed considering both the time of the half-life of aggregation ($t_{1/2}$) and the intensity of the experimental fluorescence plateau (F). The extension of $t_{1/2}$ is defined as the experimental $t_{1/2}$ in the presence of the tested compound relative to the one obtained without the compound and is evaluated as the following ratio: $t_{1/2(A\beta+\text{compound})}$ / $t_{1/2(A\beta)}$. The change of fluorescence intensity at the plateau is defined as the intensity of experimental fluorescence plateau observed with the tested compound relative to the value obtained without the compound and is evaluated as the following percentage : $(F_{A\beta+\text{compound}} - F_{A\beta})/ F_{A\beta} \times 100)$.

Table S11. Effects of compounds **G1-3, SRE1-3** on A$\beta_{1\text{-}42}$ fibrillation assessed by ThT-fluorescence spectroscopy at 10/1 and 1/1 compound/Aβ ratios (the concentration of A$\beta_{1\text{-}42}$ in this assay is 10 µM) and compared to the values obtained for A$\beta_{1\text{-}42}$ alone ($t_{1/2}$ and F) analysed at the same concentration.

Compounds (Compound/Aβ ratio)	$t_{1/2}$ extension	Change of fluorescence intensity at the plateau (%)
**G1a** (10/1)	NA	–97±1%
**G1a** (1/1)	2.06±0.12	–71±2%

**G2a** (10/1)	Sat [a]	Sat [a]
**G2a** (1/1)	1.76±0.11	–41±7%
**G1b** (10/1)	NA	–97±1%
**G1b** (1/1)	NA	–90±2%
**G2b** (10/1)	NA	–95±1%
**G2b** (1/1)	>3.56±0.12	–73±3%
**G3** (10/1)	NA	–97±1%
**G3** (1/1)	3.05±0.07	–70±4%
**SRE1** (10/1)	1.31±0.09	–26±4%
**SRE1** (1/1)	1.18±0.01	ne
**SRE2** (10/1)	ne	–19±11%
**SRE2** (1/1)	ne	–21±3%
**SRE3** (10/1)	2.72±0.07	–46±2%
**SRE3** (1/1)	1.17±0.01	ne

NA = no aggregation observed, ne = no effect, parameters are expressed as mean ± SE, n=3. [a] Sat means that a saturation of the fluorescence signal is observed because **G2a** self-aggregates at 100 μM.

(A)

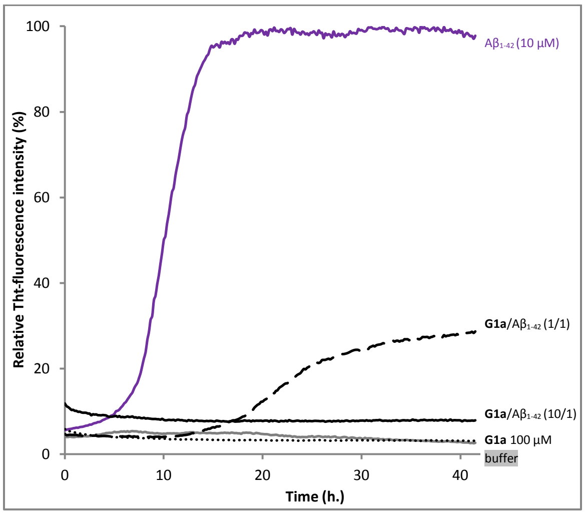

(B)

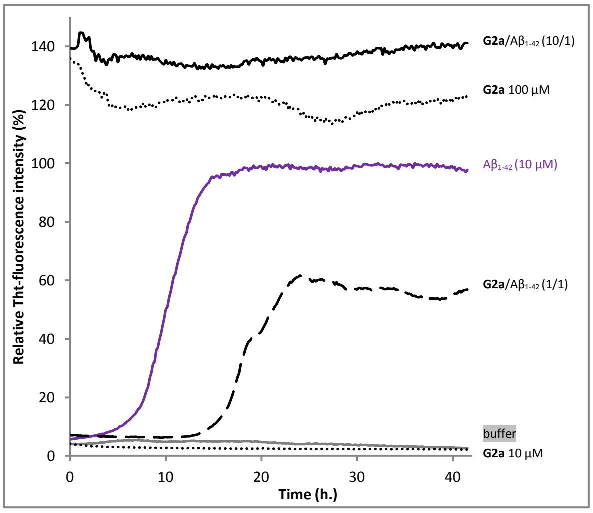

(C)

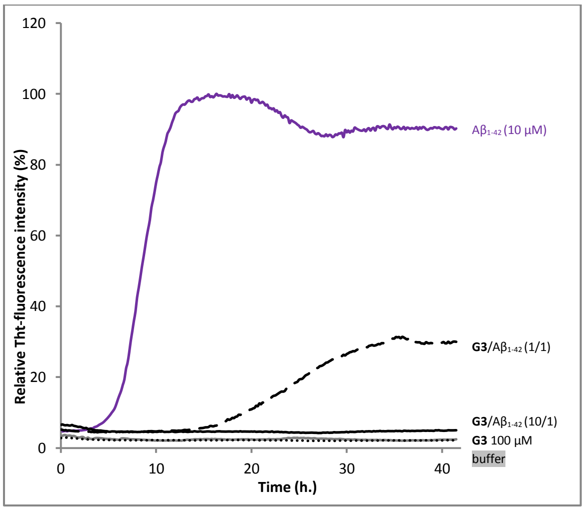

(D)

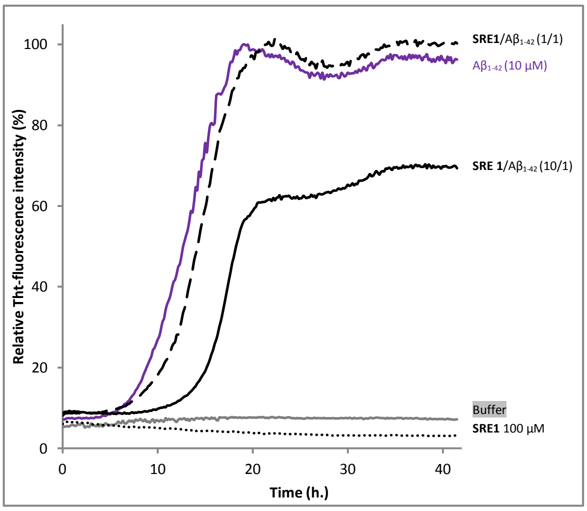

(E)

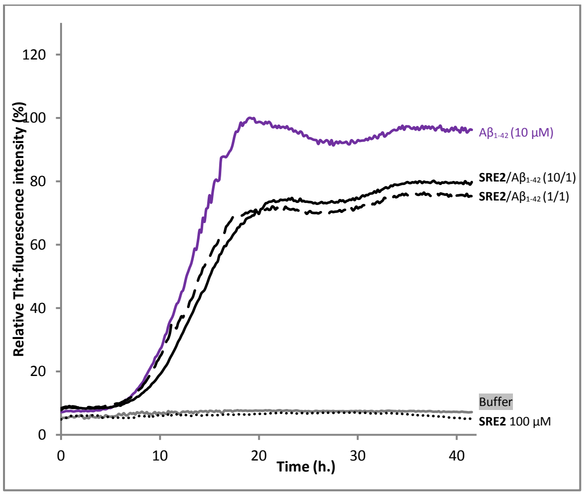

(F)

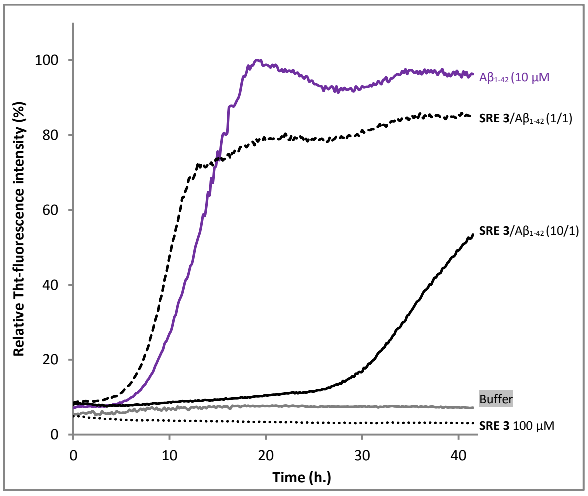

Figure S14. Representative curves of ThT-fluorescence assays over time showing $A\beta_{1-42}$ (10 μM) aggregation in the absence (purple curves) and in the presence of compounds **G1a** (A), **G2a** (B), **G3** (C), **SRE1** (D), **SRE2** (E), **SRE3** (F) at compound/$A\beta_{1-42}$ ratio of 10/1 and 1/1 (black curves).

Thioflavin-T assay (IAPP)

IAPP was purchased from Bachem. IAPP was dissolved in hexafluoro-isopropanol (HFIP) at a concentration of 1 mM and incubated for 1 hour to dissolve any preformed aggregates. Next, HFIP was evaporated with dry nitrogen gas followed by vacuum desiccation for 3 hours. The resulting peptide film was then dissolved in DMSO to obtain stock solutions of IAPP (0.2 mM). Stock solutions of compounds **G1b** and **G2b** were dissolved in DMSO (10

and 1 mM). The concentration of DMSO was kept constant at 3% (v/v) in the final volume of 200 µL.

Thioflavin-T binding assays were used to measure the development of fibrils over time. A plate reader (Fluostar Optima, BmgLabtech) and standard 96-wells flat-bottom black microtiter plates in combination with a 440 nm excitation filter and a 485 nm emission filter were used. The ThT assay was started by adding 5 µL of a 0.2 mM IAPP stock solution and 1 µL of stock solutions of compounds to test to a mixture of 10 µM ThT and 10 mM Tris/HCl, 100 mM NaCl at pH 7.4. The concentration of IAPP was held constant at 5 µM and compounds were added to yield compound/IAPP ratios of 10/1 and 1/1. The ThT assays was performed in triplicate.

It is noteworthy that both compounds **G1b** and **G2b** displayed no activity on IAPP fibrillization process at compound/A$\beta_{1\text{-}42}$ ratio of 1/1. They slightly delayed the aggregation process at the higher ratio (10/1); the $t_{1/2}$ being approximatively doubled but the fluorescence intensity at the plateau being unchanged.

(A) (B)

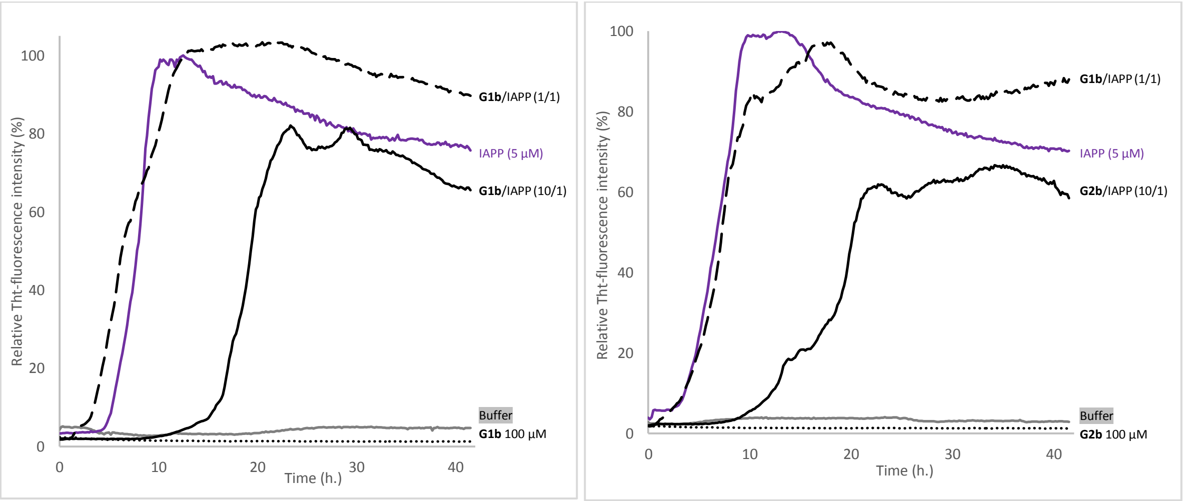

Figure S15. Representative curves of ThT-fluorescence assays over time showing IAPP (5 µM) aggregation in the absence (purple curves) and in the presence of compounds **G1b** (A), **G2b** (B) at compound/A$\beta_{1\text{-}42}$ ratio of 10/1 and 1/1 (black curves).

Transmission electron microscopy

The reduction or the increase of the final fluorescence intensity of Aβ_{1-42} induced by the peptidomimetics should not necessarily be interpreted quantitatively as the amount of fibrillar material formed. Indeed, changes in the ThT binding constant or in the quantum yield of the dye and binding of the dye to aggregated synthetic molecules might also affect the fluorescence intensity. The results of Th-T fluorescence spectroscopy has to be confirmed by TEM.

Samples were prepared under the same conditions as in the ThT-fluorescence assay. Aliquots of Aβ_{1-42} (10 µM in 10 mM Tris-HCl, 100 mM NaCl buffer, pH 7.4 in the presence and absence of the tested compounds) were adsorbed onto 300-mesh carbon grids for 2 min, washed and dried. The samples were negatively stained for 45 s. on 2 % uranyl acetate in water. After draining off the excess of staining solution and drying, images were obtained using a ZEISS 912 Omega electron microscope operating at an accelerating voltage of 80 kV.

20h 42h

Aβ_{1-42} alone

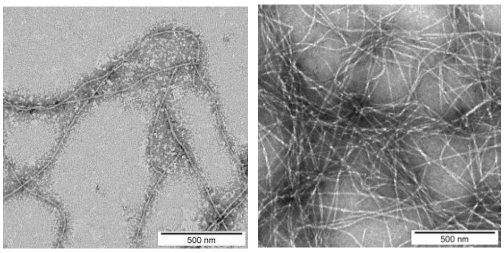

In the presence of **G1a**

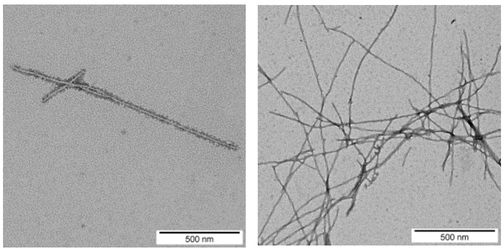

In the presence of **G1b**

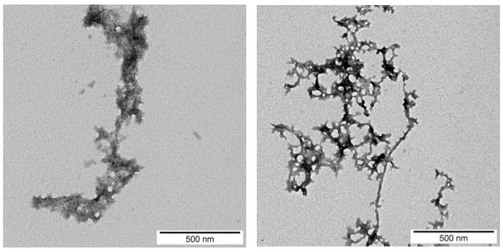

In the presence of **G2b**

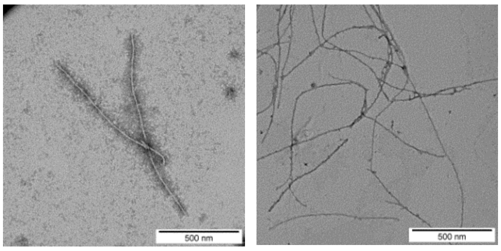

Figure S16. Effects of **G1a**, **G1b** and **G2b** derivatives on the fibril formation of Aβ_{1-42} visualized by TEM. Negatively stained images were recorded after 20 h (left row) and 42 h (right row) of incubation of Aβ_{1-42} (10 µM in 10 mM Tris.HCl, 100 mM NaCl at pH = 7.4) alone (first line) or in the presence of 10 µM of **G1a** (second line), of 10 µM of **G1b** (third line) and of 10 µM of **G2b** (fourth line). Scale bars, 500 nm.

Capillary electrophoresis

- Sample preparation: The commercial Aβ_{1-42} was dissolved upon reception in 0.16% NH_4OH (at 2 mg/mL) for 10 minutes at 20°C, followed by an immediate lyophilisation and a storage at -20°C as pretreatment.
- CE: CE experiments were carried out with a PA800 ProteomeLab instrument (Beckman Coulter Inc., Brea, CA, USA) equipped with a diode array detector. UV Detection was performed at 190 nm. The prepared sample (as previously described) was dissolved in 20 mM phosphate buffer pH 7.4 containing DMSO (control or stock solutions of compounds dissolved in DMSO) at 2.5% (v/v) and the final peptide concentration at 100 µM regardless the peptide/compound ratio.

For the CE separation of Aβ oligomers, fused silica capillary 80 cm (10.2 cm to the detector) ′ 50 mm I.D. were used. The background electrolyte was a 80 mM phosphate buffer, pH 7.4.
The separation was carried out under -30 kV at 20°C. The sample was injected from the outlet by hydrodynamic injection at 3.44 KPa for 10 s. After each run, the capillary was rinsed for 5 min with water, 1 min with SDS 50 mM, 5 min with NaOH 1 M and equilibrated with running buffer for 5 min.
We focused our attention on three kinds of species: (1) the monomer (peak ES), (2) different small metastable oligomers grouped under peak ES^{I} and (3) transient species formed later and which correspond to species larger than dodecamers and still soluble (peak LS). Aggregation kinetics of Aβ_{1-42} peptide alone (Figure S18) showed that overtime, the monomer ES peak decreased in favor of the oligomer peaks ES^{I} and LS, and that insoluble species, forming spikes in the profile, appeared after 8 hours.

In the presence of **G1b**, the aggregation kinetics of Aβ_{1-42} peptide was greatly modified (Figure S19). Noteworthy, the monomeric species (peak ES) was dramatically stabilized. 86% of the monomer remained after 24 h in the presence of **G1b**, while it was no more detected in the control sample. Moreover, the larger aggregated species LS (> dodecamers) were not detected but new aggregated forms of Aβ_{1-42}, between ES' and LS migration times were observed on each electrophoretic profile. We checked that these new aggregated forms of Aβ_{1-42} were not due to **G1b** degradation or self-assemblies (Figure S17A.) They were probably aggregated forms with a different morphology than both LS and those giving spikes observed in Aβ_{1-42} control. This observation is in accordance with the TEM images where

globular aggregates were observed instead of the classical dense network of fibers (Figures 5c) and S16). In ThT-assays, no fluorescence was detected, indicating that the globular species were not characterized by a highly ordered β-structures (Figure 5a)). Considering the intensities of ES and ES^{I} peaks over time it seems that **G1b** induces the formation of metastable species at the beginning of the kinetics that are then transformed into monomeric state (Figures S19 and S21). Remarkably, the presence of the monomer was maintained even after 4 days (Figure S19B). We concluded that **G1b** is able to prevent the formation of toxic soluble oligomers of $A\beta_{1-42}$ peptide and to maintain the presence of the non toxic monomer overtime.

The electrophoretic profile of $A\beta_{1-42}$ in the presence of **G2b** (**G2b**/$A\beta_{1-42}$ ratio of 1/1) was very different from the one observed in the presence of **G1b**. The oligomerization process was dramatically delayed in comparison with the kinetics control (Figure S20). **G2b** also dramatically maintained the presence of the monomer (peak ES). 80% of the monomer remained after 24 h (Figure 6c) and S21). New aggregated forms were only transiently observed (after 8h) but were not longer detectable after 24h. This result was also in accordance with the TEM images where we observed a much less dense network of fibers, however the typical morphology was retained.

A)

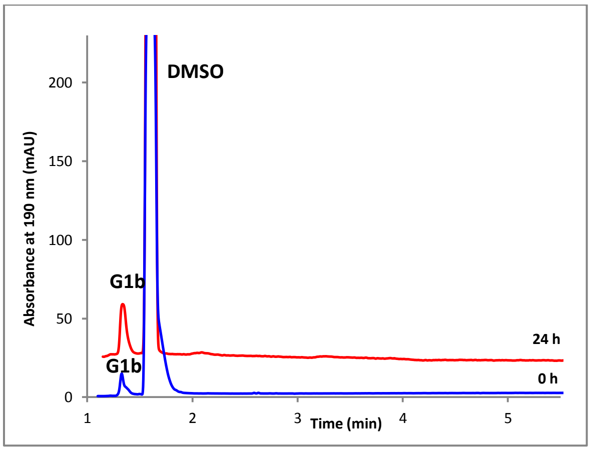

B)

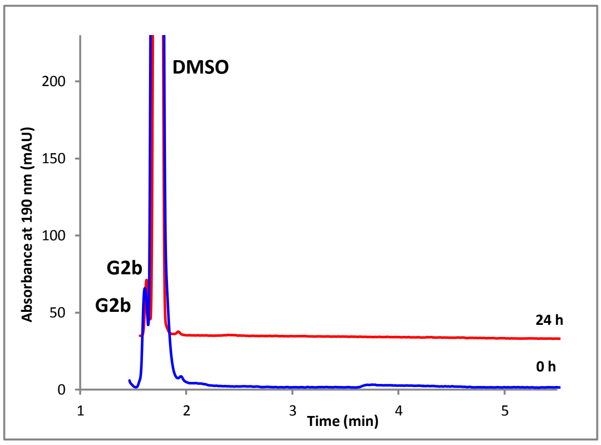

Figure S17. Electrophoretic profiles of **G1b** (A) and **G2b** (B) alone at 0 h (blue) and 24 h (red) in phosphate buffer 20 mM and DMSO (2.5%), showing that no extra peak appeared during the incubation of these coupounds.

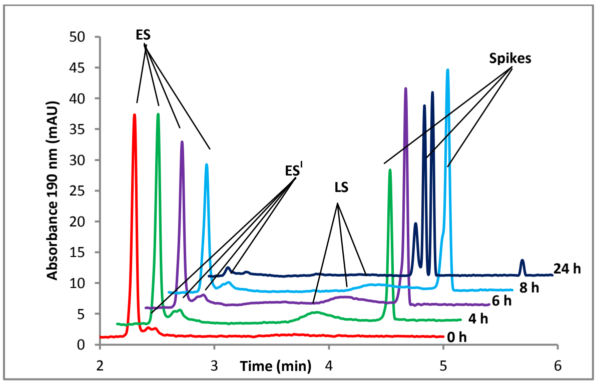

Figure S18. Electrophoretic profile of Aβ$_{1-42}$ peptide (100 µM) at 0 h (red), 4 h (green), 6 h (purple), 8 h (light blue) and 24 h (blue) in phosphate buffer 20 mM and DMSO (2.5 %). The monomeric species at 2.3 min decreases dramatically overtime.

A) B)

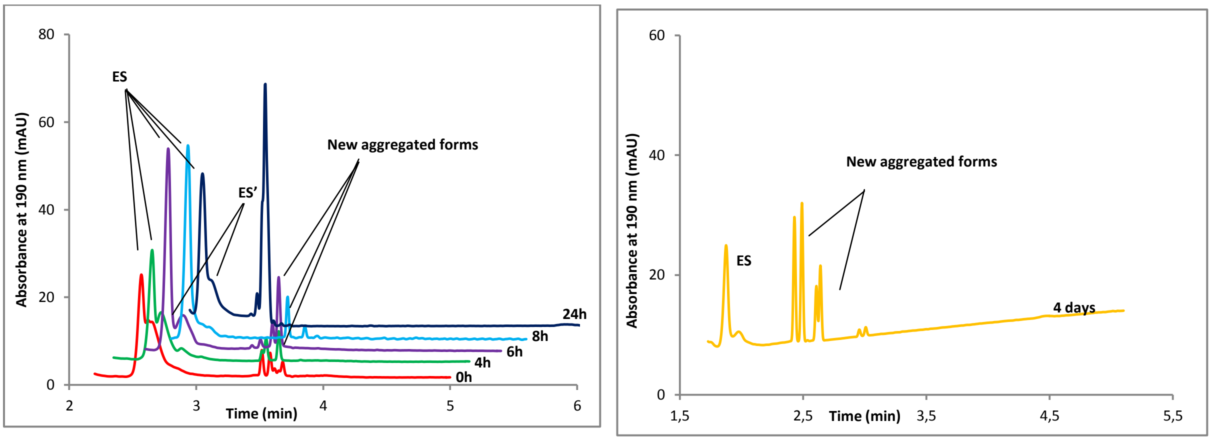

Figure S19. Electrophoretic profile of Aβ$_{1-42}$ peptide (100 µM) in the presence of **G1b** at a **G1b**/Aβ$_{1-42}$ ratio of 1/1 at 0 h (red), 4 h (green), 6 h (purple), 8 h (light blue), 24 h (blue) and 4 days (yellow) in phosphate buffer 20 mM and DMSO (2.5%).

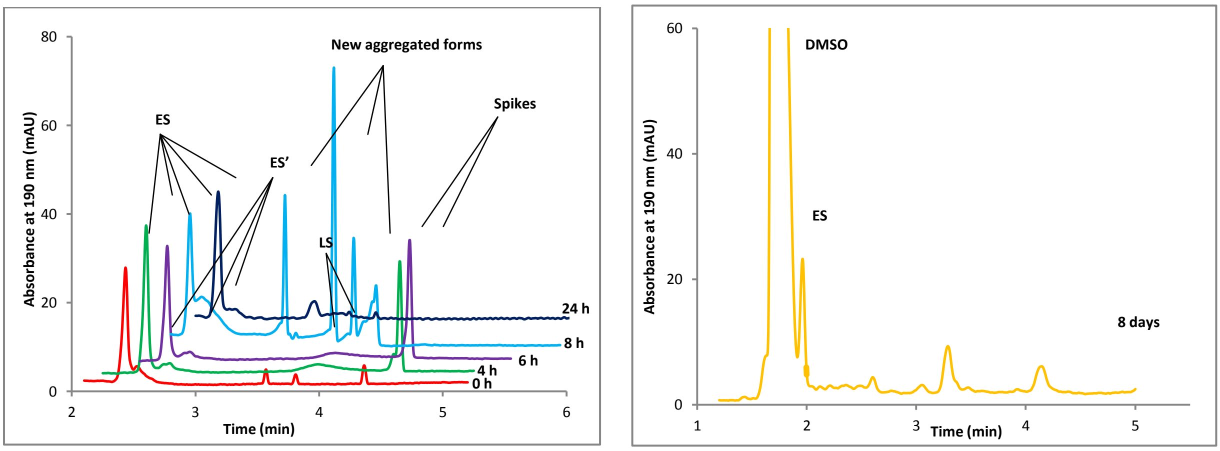

Figure S20. Electrophoretic profile of Aβ$_{1-42}$ peptide in the presence of **G2b** at a **G2b**/Aβ$_{1-42}$ ratio of 1/1 at 0 h (red), 6 h (green), 8 h (purple), 16 h (light blue), 24 h (blue) and 8 days (yellow) in phosphate buffer 20 mM and DMSO (2.5%).

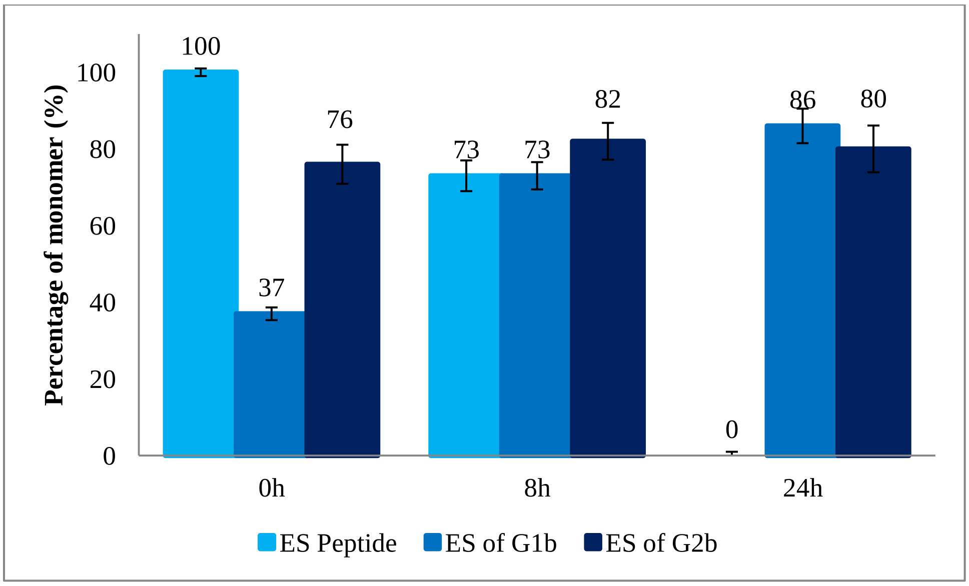

Figure S21. Peak area of the monomer in **G1b** and **G2b** profiles at 0, 8 and 24 h related to the peak area of Aβ$_{1-42}$ peptide control at 0h. Results are a mean of 3 experiments

Cell toxicity

SH-SY5Y neuroblastoma cells were grown in low serum Optimem (Life Technologies) for 24 hours at 37˚C, 5% CO2 in a 96 well plate at 20 000 cells per well. Aβ$_{1-42}$ was dissolved in sterile PBS at 50 µM concentration in the presence of 1, 5, 10 and 50 µM of the four compounds for 24 hours at room temperature, along with a control incubation with no inhibitor. After the 24 hour period, media was removed from the cells and replaced with Optimem containing the pre-incubated Aβ$_{1-42}$ plus inhibitor diluted one in ten (5 µM Aβ final concentration) in quadruplicate. The cells were incubated for a further 24 hours as before and the cell viability (MTS assay) and cell proliferation (LDH assay) assessed using the CellTiter 96® Aqueous One Solution Cell Proliferation Assay (Promega) and CytoTox 96® Non-Radioactive Cytotoxicity Assay (Promega) respectively. The assays were repeated twice and representative samples are shown.

Bibliography

[1] S. Pellegrino, A. Contini, F. Clerici, A. Gori, D. Nava, M. L. Gelmi, *Chem. – A Eur. J.* **2012**, *18*, 8705–8715.

[2] A. Ruffoni, A. Contini, R. Soave, L. Lo Presti, I. Esposto, I. Maffucci, D. Nava, S. Pellegrino, M. L. Gelmi, F. Clerici, *RSC Adv.* **2015**, *5*, 32643–32656.

[3] Molecular Operating Environment (MOE), Chemical Computing Group Inc., Montreal, 2013.

[4] E. D. C. Cézard, E. Vanquelef, J. Pecher, P. Sonnet, P. Cieplak, F.-Y. Dupradeau, in 236th ACS Natl. Meet. Philadelphia, PA, USA, August 17, 2008.

[5] D. A. Case, V. Babin, J. T. Berryman, R. M. Betz, Q. Cai, D. S. Cerutti, I. T.E. Cheatham, T. A. Darden, R. E. Duke, H. Gohlke, *et al.*, AMBER 14, University of California, San Francisco, 2014.

[6] P. A. Kollman, *Acc. Chem. Res.* **1996**, *29*, 461–469.

[7] A. Onufriev, D. Bashford, D. A. Case, *Proteins Struct. Funct. Bioinform.* **2004**, *55*, 383–394.

[8] A. Patriksson, D. van der Spoel, *Phys. Chem. Chem. Phys.* **2008**, *10*, 2073–2077.

[9] J.-P. Ryckaert, G. Ciccotti, H. J. . Berendsen, *J. Comput. Phys.***1977**, *23*, 327–341.

[10] W. Kabsch, C. Sander, *Biopolymers* **1983**, *22*, 2577–2637.

[11] T. Caliński, J. Harabasz, *Commun. Stat.* **1974**, *3*, 1–27.

[12] S. Pellegrino, A. Contini, M. L, Gelmi, L. Lo Presti, R. Soave, E. Erba *J. Org. Chem.* **2014**, *79*, 3094−3102)

[13] S. Pellegrino, C. Annoni, A. Contini, F. Clerici, M. L. Gelmi *Amino Acids* **2012**, *43*, 1995-2003.

[14] D. S. King, C. G. Fields, G. B. Fields *International Journal of Peptide and Protein Research* **1990**, *36*, 255–266)

[15] P. Cuniasse, I. Raynal, A. Yiothakis, V. Dive *J. Am. Chem. Soc.* **1997**, *119*, 5239-5248.

[16] D. S. Wishart, B**.** D. Sykes, F. M. Richards *Biochemistry* **1992,** *31*, 1647-1651.

[17] A. M. Fernandez-Escamilla, S. Ventura, L. Serrano, M. A. Nez, *J. Prot. Sci.* **2006**, *15*, 2278-2289.

[18] D. S. Wishart, C.G. Bigam, A. Holm, R. S. Hodges, B. D. Sykes *J. Biomol. NMR* **1995** *5* 67-81.

[19] C. D. Tatko, M. L. Waters *J. Am. Chem. Soc.* **2002**, *124*, 9373

[20] a) Zerella, R. *Protein Sciences* **1999**, *8*, 1320-1331; b) Smith, L.J.; Bolin K.A., Schwalbe, H., Macarthur, M.W., Thornton, J.M., Dobson CM. 1996. *J Mol Biol* **1996**, *255*, 494-506.
[21] L. De Rosa, D. Diana, A. Basile, A. Russomanno, C. Isernia, M. C. Turco, R. Fattorusso, L. D. D'Andrea *Eur. J. Med. Chem.* **2014**, *73*, 210-216
